# Spatially interpretable artificial intelligence framework to tailored neoadjuvant dual HER2 blockade in HER2-positive breast cancer

**DOI:** 10.1038/s41392-026-02734-0

**Published:** 2026-06-19

**Authors:** Xiang-Rong Wu, Hong Lv, Shen Zhao, Xiao-Hua Zeng, Lei-Jie Dai, Yu-Zheng Xu, Yu-Wei Li, Zi-Yu Qiu, Ji-Ting Huang, Ning-Ning Zhang, Li Chen, Min He, Yi-Zhi Zhao, Lin Yang, Tong Zhou, Jun-Jie Li, Jiong Wu, Yi-Zhou Jiang, Wen-Tao Yang, Gen-Hong Di, Zhi-Ming Shao, Ding Ma

**Affiliations:** 1 Department of Breast Surgery, Key Laboratory of Breast Cancer in Shanghai, Fudan University Shanghai Cancer Center, Shanghai, 200032 China; 2 Department of Oncology, Shanghai Medical College, Fudan University, Shanghai, 200032 China; 3 Department of Pathology, Fudan University Shanghai Cancer Center, Shanghai, 200032 China; 4 Department of Breast Cancer Center, Chongqing University Cancer Hospital, Chongqing, 400030 China; 5 School of Engineering, Westlake University, Hangzhou, 310030 China; 6 Hangzhou DiPath Technology Co. Ltd., Hangzhou, 311121 China; 7 Shanghai Institute of Preventive Medicine, Shanghai, 201416 China; 8 Shanghai Academy of Natural Sciences (SANS), Fudan University, Shanghai, 200030 China

**Keywords:** Predictive medicine, Tumour biomarkers, Breast cancer, Imaging, Predictive markers

## Abstract

Neoadjuvant dual HER2 blockade with trastuzumab and pertuzumab plus chemotherapy represents the current standard-of-care for HER2-positive breast cancer. However, treatment responses remain heterogeneous, underscoring the lack of clinically practical tools for predicting treatment efficacy and informing personalized therapy. Here, we developed HER2-LADDER (Layered AI-based Dual-targeteD anti-HER2 Recommendation), a spatially interpretable and clinically accessible artificial intelligence framework that integrates clinicopathological and spatial topological features from routine hematoxylin and eosin (H&E) and HER2 immunohistochemistry (IHC) slides. Using these spatially derived features, HER2-LADDER accurately predicted response to neoadjuvant TCbHP/PCbHP, achieving AUCs of 0.944 in the model construction cohort (*N* = 276), 0.917 in the temporal validation cohort (*N* = 82), and 0.869 in the trial-based validation cohort (*N* = 85). On the basis of HER2-LADDER scores, patients were stratified into Low (highly responsive), Medium (responsive), and High (resistant) groups, identifying candidates for treatment de-escalation (THP or TCbH/PCbH), standard-of-care (TCbHP/PCbHP), or alternative regimens (e.g., next-generation anti-HER2 antibody‐drug conjugates), respectively. Importantly, Xenium in situ profiling further revealed biological correlates underlying model predictions, including HER2-enriched tumor cell aggregation and neutrophil-helper T-cell interactions, thereby highlighting the mechanistic interpretability of the model. Collectively, HER2-LADDER unites digital pathology and high-resolution spatial profiling into a clinically accessible AI framework, offering a robust, transparent, and biologically grounded tool to tailor individualized HER2-targeted therapy optimization.

## Introduction

Human epidermal growth factor receptor 2-positive (HER2+) breast cancer accounts for ~15–20% of all breast cancer subtypes and is characterized by aggressive behavior and poor prognosis.^1,2^ Biologically, HER2 is a member of the ErbB receptor tyrosine kinase family. When overexpressed or amplified, HER2 promotes receptor dimerization and activates downstream pathways such as PI3K/AKT and MAPK, thereby enhancing tumor-cell proliferation, survival, and invasion.^3,4^ Although such activation drives tumor progression and contributes to worse clinical outcomes, this biological dependency also makes HER2 an actionable therapeutic target. Monoclonal antibodies such as trastuzumab can inhibit HER2-driven signaling and mediate antibody-dependent cytotoxicity, fundamentally changing the natural history of this disease.^5^ The clinical development of anti-HER2 therapy has therefore reshaped the treatment paradigm for HER2-positive breast cancer. Compared with chemotherapy alone, the advent of trastuzumab markedly reduced the recurrence risk and improved overall survival (OS).^6,7^ In the neoadjuvant setting, the NOAH trial demonstrated that incorporating trastuzumab into chemotherapy improved 3-year event-free survival from 56 to 71% in patients with HER2-positive locally advanced or inflammatory breast cancer.^8^ Subsequently, dual HER2 blockade further improved neoadjuvant efficacy. In the NeoSphere trial, the addition of pertuzumab to trastuzumab and docetaxel increased the pathological complete response (pCR) rate from 29.0 to 45.8%.^9^ PEONY further confirmed this benefit in an Asian population.^10,11^ Then, a series of clinical trials optimized the chemotherapy backbone, and laid the groundwork for the standard regimens such as platinum-taxane dual-targeted regimens (TCbHP/PCbHP), which are now widely recommended in clinical guidelines worldwide.^2,12–14^

Despite these therapeutic advances, treatment response remains markedly heterogeneous across patients with HER2-positive breast cancer.^15^ Clinically, this heterogeneity creates a two-sided challenge: some patients may maintain excellent outcomes with less intensive strategies, including chemotherapy de-escalation,^16–18^ whereas others show a suboptimal response despite standard therapy and may eventually require treatment adaptation, including next-generation antibody-drug conjugates (ADCs)^4,14^ or tyrosine kinase inhibitors (TKIs).^19,20^ These clinical realities make pretreatment response stratification increasingly important, yet robust and clinically actionable tools remain limited. Existing predictive approaches predominantly rely on biomarkers such as HER2 amplification status^21^ and CD8-positive tumor-infiltrating lymphocytes (CD8+ TILs).^22,23^ Although these biomarkers are informative, they lack sufficient granularity and precision to effectively distinguish responders from nonresponders. Additionally, sequencing-based predictive assays,^24–28^ while promising, may face substantial practical limitations, including high costs and limited clinical accessibility, which modestly restrict their widespread clinical implementation.^29^ Taken together, these limitations highlight the need for clinically accessible approaches that can better capture treatment-relevant tumor biology.^30^

One practical route toward this goal is to leverage routine pathology slides, which are already generated in standard care and preserve rich information on cell morphology, microenvironmental composition, and tissue architecture.^31^ Recent work has shown that artificial intelligence applied to histopathology can recover clinically relevant biological signals that were previously thought to require dedicated molecular assays. For example, a recent multicenter study demonstrated that deep learning on histopathological images, combined with clinicopathological variables, could estimate Oncotype DX recurrence risk and chemotherapy benefit, supporting the broader concept that routine pathology can serve as a surrogate substrate for treatment-relevant biomarker discovery.^32^ Our previous study, which investigated biomarkers of response to a next-generation ADC, SHR-A1811, further suggested that integrating baseline hematoxylin and eosin (H&E) morphology with HER2 immunohistochemistry (IHC) features could predict response to anti-HER2 ADC therapy.^33^ HER2 IHC provided information on both target expression intensity and its spatial distribution, whereas H&E captured complementary features of cell morphology, tissue architecture, and the surrounding microenvironment. This multimodal integration may therefore better reflect treatment-relevant tumor biology than either modality alone.^33^ While that work had clear boundaries: it was developed and validated in a relatively small cohort, and its clinical scope was limited to ADC response. Nevertheless, it did inspire us that such a scalable and clinically applicable framework could also be applied to standard dual HER2 blockade to achieve more precise therapeutic decision-making.

Against this background, we developed HER2-LADDER (Layered AI-based Dual-targeteD anti-HER2 Recommendation), a digital pathology framework that integrates routine clinicopathological variables with spatially resolved morphological and topological features extracted from baseline H&E and HER2 IHC whole-slide images (WSIs). By leveraging routinely available biopsy specimens, this approach enables spatially informed and clinically accessible prediction of treatment response. In this study, we established and validated HER2-LADDER across real-world and clinical trial cohorts and demonstrated robust predictive performance and generalizability. Beyond risk prediction, the framework provides layered treatment recommendations aligned with contemporary de-escalation and consideration of alternative regimens. By linking spatial tumor organization to therapeutic outcomes using standard pathology slides, our work introduces a biologically interpretable and scalable strategy to support precision decision-making in patients with HER2-positive breast cancer.

## Results

### Patient samples, clinical data, and study cohorts

**Fig. 1:**
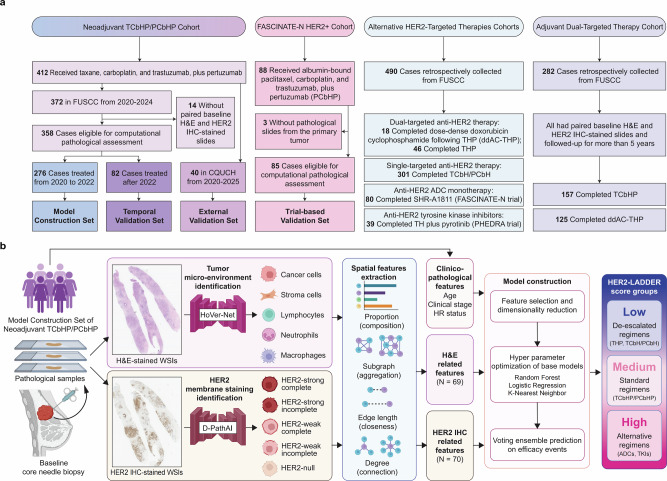
Cohort description and HER2-LADDER construction workflow. **a** Overview of the study cohorts, including patient distribution, treatment regimens, and stratification criteria of the model development cohort and validation sets. **b** Workflow for HER2-LADDER model development. Baseline core-needle biopsies (CNBs) were subjected to digital scanning of paired hematoxylin and eosin (H&E) and HER2 immunohistochemistry (IHC) slides. The HoVer-Net and D-PathAI algorithms segmented tumor–microenvironment cells (cancer cells, stromal cells, lymphocytes, neutrophils, and macrophages) and tumor cell HER2 expression (strong/weak/null with integrity of membrane staining), respectively. Spatial features were extracted via single-cell morphological and topological profiling (sc-MTOP). Selected features and clinicopathological factors (age, clinical stage, and hormone receptor status) were integrated through multimodal ensemble voting to establish the HER2-LADDER predictive score. Created with BioRender.com

To ensure robust development, external validation, and clinical applicability of the model, we systematically integrated multiple distinct cohorts representing a range of treatment scenarios and clinical settings, ultimately including a total of 1,249 patients with HER2-positive breast cancer for analysis (Fig. 1a and Table 1).

**Table 1:** Baseline clinicopathological characteristics of the cohorts included in the study

Treatment cohort	*N*	Age at diagnosis (mean [SD])	Efficacy (%)		HR status (%)		cT stage (%)			cN stage (%)				HER2 IHC score (%)	
			pCR	non-pCR	Negative	Positive	≤2	3	4	0	1	2	3	2 (FISH + )	3
Model construction															
TCbHP/PCbHP	276	50.9 (10.7)	175 (63.4%)	101 (36.6%)	147 (53.3%)	129 (46.7%)	197 (71.4%)	44 (15.9%)	35 (12.7%)	27 (9.8%)	151 (54.7%)	54 (19.6%)	44 (15.9%)	32 (11.6%)	244 (88.4%)
Temporal validation															
TCbHP/PCbHP	82	50.2 (9.9)	53 (64.6%)	29 (35.4%)	38 (46.3%)	44 (53.7%)	63 (76.8%)	12 (14.6%)	7 (8.5%)	7 (8.5%)	39 (47.6%)	24 (29.3%)	12 (14.6%)	9 (11.0%)	73 (89.0%)
FASCINATE-N HER2-positive															
PCbHP	85	50.5 (11.0)	56 (65.9%)	29 (34.1%)	55 (64.7%)	30 (35.3%)	49 (57.6%)	18 (21.2%)	18 (21.2%)	5 (5.9%)	35 (41.2%)	25 (29.4%)	20 (23.5%)	12 (14.1%)	73 (85.9%)
CQUCH external validation															
TCbHP/PCbHP	40	52.5 (7.3)	21 (52.5%)	19 (47.5%)	20 (50.0%)	20 (50.0%)	27 (67.5%)	2 (5.0%)	11 (27.5%)	2 (5.0%)	14 (35.0%)	12 (30.0%)	12 (30.0%)	5 (12.5%)	35 (87.5%)
Alternative neoadjuvant															
ddAC-THP	18	47.7 (11.4)	9 (50.0%)	9 (50.0%)	9 (50.0%)	9 (50.0%)	10 (55.6%)	6 (33.3%)	2 (11.1%)	2 (11.1%)	8 (44.4%)	6 (33.3%)	2 (11.1%)	3 (16.7%)	15 (83.3%)
THP	46	55.4 (12.2)	21 (45.7%)	25 (54.3%)	30 (65.2%)	16 (34.8%)	31 (67.4%)	9 (19.6%)	6 (13.0%)	3 (6.5%)	24 (52.2%)	13 (28.3%)	6 (13.0%)	2 (4.3%)	44 (95.7%)
TCbH/PCbH	301	50.5 (10.1)	134 (44.5%)	167 (55.5%)	156 (51.8%)	145 (48.2%)	194 (64.5%)	68 (22.5%)	39 (13.0%)	40 (13.3%)	152 (50.5%)	55 (18.3%)	54 (17.9%)	39 (13.0%)	262 (87.0%)
SHR-A1811	80	47.8 (10.1)	49 (61.3%)	31 (38.8%)	41 (51.2%)	39 (48.8%)	51 (63.7%)	9 (11.2%)	20 (25.0%)	8 (10.0%)	23 (28.7%)	21 (26.2%)	28 (35.0%)	9 (11.2%)	71 (88.8%)
TH+Pyrotinib	39	50.4 (9.3)	21 (53.8%)	18 (46.2%)	21 (53.8%)	18 (46.2%)	37 (94.9%)	2 (5.1%)	0 (0.0%)	0 (0.0%)	4 (10.3%)	34 (87.2%)	1 (2.6%)	0 (0.0%)	39 (100.0%)
Real-world adjuvant															
TCbHP	157	50.8 (10.0)	–	–	77 (49.0%)	80 (51.0%)	145 (92.4%)	12 (7.6%)	0 (0.0%)	127 (80.9%)	8 (5.1%)	15 (9.6%)	7 (4.5%)	11 (7.0%)	146 (93.0%)
ddAC-THP	125	48.2 (9.5)	–	–	52 (41.6%)	73 (58.4%)	118 (94.4%)	7 (5.6%)	0 (0.0%)	87 (69.6%)	16 (12.8%)	16 (12.8%)	6 (4.8%)	12 (9.6%)	113 (90.4%)

As the core datasets for model development and validation, the cohort included a total of 372 HER2-positive breast cancer patients who completed standard-of-care taxane, carboplatin, trastuzumab, and pertuzumab (TCbHP/PCbHP) regimens at Fudan University Shanghai Cancer Center (FUSCC) between 2020 and 2024. After 14 patients without paired baseline H&E and HER2 IHC slides were excluded, 358 patients were eligible for computational pathological analysis. Among them, 276 cases treated from 2020 to 2022 constituted the model construction set, while 82 cases treated after 2023 formed a temporal validation set to assess the reproducibility of HER2-LADDER in more recent real-world patients. The FASCINATE-N trial (NCT05582499) HER2-positive cohort comprised 88 patients treated with PCbHP between December 2022 and February 2024.^19^ After three cases without available slides of the primary tumor were excluded, 85 patients were included as a trial-based validation set for independent evaluation of HER2-LADDER in a prospective clinical trial setting. In addition, an independent multicenter external validation cohort from Chongqing University Cancer Hospital (CQUCH) included 40 HER2-positive patients treated with neoadjuvant TCbHP/PCbHP, with paired baseline H&E and HER2 IHC slides available for analysis. Across these cohorts, patients exhibited broadly similar baseline characteristics, with median ages between 50.2 and 52.5 years.

To further explore the applicability of the model in nonstandard or adaptive therapeutic scenarios, 490 cases were retrospectively collected from patients treated at FUSCC. A total of 484 patients in these cohorts had paired baseline H&E and HER2 IHC slides available for spatial feature extraction. These included 18 patients who received dose-dense doxorubicin–cyclophosphamide followed by THP (ddAC-THP) and 46 who received THP directly under dual-targeted regimens; 301 patients who received single-targeted regimens with TCbH/PCbH; 80 patients who received SHR-A1811 (trastuzumab rezetecan), a next-generation anti-HER2 ADC composed of a humanized HER2-directed monoclonal antibody, a cleavable tetrapeptide linker, and a DNA topoisomerase I inhibitor payload (FASCINATE-N trial, NCT05582499)^19,33^; and 39 patients who received docetaxel, trastuzumab plus the pan-ErbB TKI pyrotinib (PHEDRA trial, NCT03588091).^20^

Finally, to assess the prognostic value of the model in the adjuvant setting, we analyzed a separate cohort of patients from FUSCC who received dual-targeted chemotherapy after surgery between 2018 and 2020. Among them, 157 received TCbHP, and 125 received ddAC-THP, with follow-up exceeding 5 years, to evaluate long-term outcomes, including OS and disease-free survival (DFS).

### Establishment of the HER2-LADDER model

The HER2-LADDER model was developed using real-world HER2-positive breast cancer cohorts treated with standard neoadjuvant TCbHP/PCbHP. Paired H&E and HER2 IHC slides from these biopsies underwent digital scanning. Two specialized deep-learning algorithms were subsequently employed for detailed single-cell segmentation (Fig. 1b): HoVer-Net distinguished components of the tumor–microenvironment—including tumor cells, stromal cells, lymphocytes, neutrophils, and macrophages—on H&E slides, while D-PathAI characterized tumor cells on HER2 IHC slides according to HER2 membrane staining intensity and completeness.

Following cellular segmentation, a single-cell morphological and topological profiling (sc-MTOP) framework was applied to systematically extract spatially resolved single-cell features. This procedure generated a total of 69 features from H&E images and 70 from HER2 IHC images, encompassing cell proportions, spatial interactions, and cellular distributions. As summarized in Supplementary Table 1, these spatial features were broadly classified into three functional categories: (i) proportion, which represents the compositional ratios of specific cell types within the tissue; (ii) Nsubgraph, which indicates the number of cells within defined local cellular clusters, reflecting aggregation tendencies; (iii) edge-length metrics (MinEdgeLength and MeanEdgeLength), which quantify the spatial distance between specific cell types; and (iv) degree, which captures the connectivity and centrality of individual cells within their immediate cellular neighborhoods.

These digital pathology-derived spatial features were subsequently integrated with critical clinical-pathological variables, including patient age, clinical stage, and hormone receptor (HR) status. Through systematic data splitting, feature engineering, multimodel hyperparameter tuning, and ensemble voting, we finalized the HER2-LADDER predictive scoring model (Supplementary Fig. 1). Model performance, generalizability, and clinical utility were subsequently validated across multiple real-world and trial-based patient cohorts.

### Predictive performance of the HER2-LADDER model in neoadjuvant settings

**Fig. 2:**
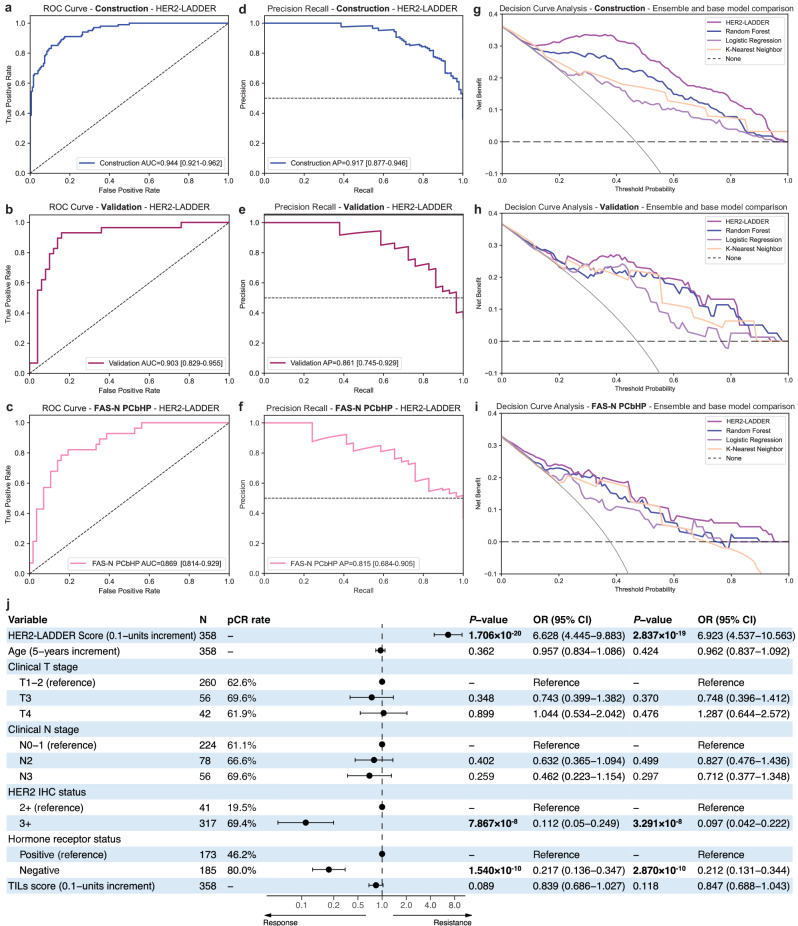
Comprehensive evaluation of the efficacy prediction performance of the HER2-LADDER model. **a**–**c** Receiver operating characteristic (ROC) curve illustrating HER2-LADDER model performance in the model construction (**a**) (AUC = 0.944), temporal validation (**b**) (AUC = 0.903), and FASCINATE-N PCbHP (trial-based validation) (**c**) (AUC = 0.869) cohorts. **d**–**f** Average precision (AP) curve of the HER2-LADDER model in the model construction (**a**) (AP = 0.917), temporal validation (**b**) (AP = 0.861), and FASCINATE-N PCbHP validation (**c**) (AP = 0.815) cohorts. **g**–**i** Decision curve analysis (DCA) highlighting the clinical net benefit of the ensemble voting model compared with that of individual submodels across the model construction (**g**), temporal validation (**h**), and FASCINATE-N PCbHP (**i**) cohorts. **j** Forest plot of logistic regression analysis showing odds ratios (ORs) for predicting treatment resistance for HER2-LADDER score (per 0.1-unit increment) and traditional clinical-pathological factors; the multivariate model was performed on the combined model construction set (*N* = 276) and temporal validation set (*N* = 82) and was adjusted for age and clinical stage

Following the modeling workflow detailed in Fig. 1b, the HER2-LADDER model demonstrated robust and consistent predictive performance across both the construction and validation sets. Specifically, the area under the curve (AUC) of the model was 0.944 (95% CI, 0.921–0.962) in the model construction set (Fig. 2a), 0.903 (95% CI, 0.829–0.955) in the independent temporal validation set (Fig. 2b), and 0.869 (95% CI, 0.814–0.929) in the FASCINATE-N PCbHP set (Fig. 2c). Similarly, the average precision (AP) scores, which reflect the model’s discriminative power in imbalanced classification settings, were 0.917 (95% CI, 0.877–0.946) for model construction (Fig. 2d), 0.861 (95% CI, 0.745–0.929) for independent temporal validation (Fig. 2e), and 0.815 (95% CI, 0.684–0.905) for FASCINATE-N PCbHP validation. Decision curve analysis (DCA) further demonstrated that the voting ensemble model consistently outperformed its individual submodel components in both cohorts, highlighting the superior net clinical benefit and the advantage of integrative modeling approaches (Fig. 2g–i). Confusion matrix analyses demonstrated consistent classification accuracy and reproducibility across cohorts, with Cohen’s kappa coefficients ≥0.76 (Supplementary Fig. 2a–c).

In addition, HER2-LADDER achieved a sensitivity of 0.8716 and specificity of 0.8933 in the model construction set, 0.8966 and 0.8600 in the temporal validation set, and 0.8173 and 0.8947 in the trial-based validation set, indicating balanced discrimination across cohorts. Calibration curve analysis further confirmed the agreement between the predicted and observed probabilities across all cohorts (Supplementary Fig. 2d). The Brier scores were 0.117 for the model construction set, 0.150 for the temporal validation set, and 0.155 for the FASCINATE-N PCbHP cohort, all of which are indicative of reliable probabilistic calibration. In an independent real-world cohort treated with the neoadjuvant ddAC-THP regimen, the discriminative performance of the model was stable, with an AUC of 0.852 and a Cohen’s kappa of 0.66 (Supplementary Fig. 2e, f). Furthermore, in the independent CQUCH cohort, HER2-LADDER achieved an AUC of 0.798, demonstrating preserved discriminative performance when extrapolated to an external medical center (Supplementary Fig. 3). These results indicate stable model behavior and support the generalizability of the predictive framework.

The predictive strength of the HER2-LADDER score was further supported by logistic regression analyses conducted on the combined set across 358 real-world neoadjuvant TCbHP/PCbHP patients (Fig. 2j). Each 0.1-unit increase in the HER2-LADDER score increased the odds of a nonpathological complete response (non-pCR) after TCbHP/PCbHP treatment nearly sevenfold (OR 6.628, 95% CI: 4.445–9.883, *P* = 1.706 × 10^−20^), outperforming conventional clinicopathologic predictors such as HER2 IHC 3+ status (OR 0.112, 95% CI: 0.05–0.249, *P* = 7.867 × 10^−8^), HR negativity (OR 0.217, 95% CI: 0.126–0.347, *P* = 1.540 × 10^−10^), and TILs score (OR 0.839, 95% CI: 0.686–1.027, *P* = 0.089). This association remained robust in the multivariable logistic regression after adjusting for age and clinical stage (adjusted OR 6.923, 95% CI: 4.537–10.563; *P* = 2.837 × 10^−19^). We further compared the discrimination ability of HER2-LADDER with that of conventional clinicopathologic variables using AUC and DeLong tests (Supplementary Fig. 4). Compared with individual clinicopathologic predictors, HER2-LADDER consistently achieved higher AUCs, with DeLong tests confirming its statistically significant superiority across all cohorts and demonstrating the superior and consistent predictive value of HER2-LADDER over traditional clinicopathologic factors. Together, these results highlight the strong predictive performance, clinical robustness, and potential of the model to inform neoadjuvant treatment decisions involving trastuzumab- and pertuzumab-based dual HER2 blockade combined with chemotherapy.

### Layered treatment recommendation and optimization informed by the HER2-LADDER model

To examine the potential clinical applicability of the HER2-LADDER model across distinct anti-HER2 therapeutic scenarios, we evaluated its predictive associations in neoadjuvant cohorts receiving different treatment regimens.

**Fig. 3:**
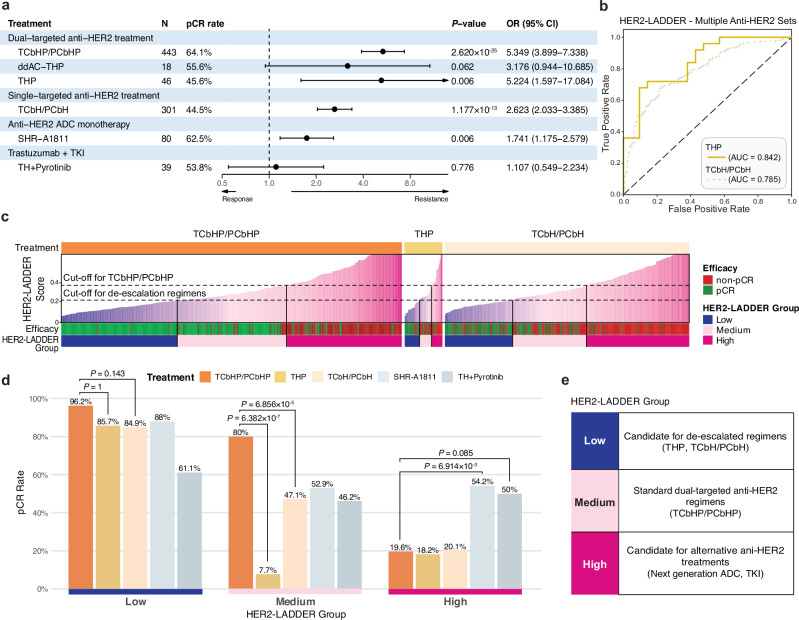
Layered stratification by the HER2–LADDER model informs treatment de-escalation or alternative strategies. **a** Logistic regression analysis demonstrating significant correlations between the HER2-LADDER score and therapeutic outcomes across diverse dual-targeted (TCbHP/PCbHP, ddAC-THP, and THP), single-targeted (TCbH/PCbH), and alternative anti-HER2 regimens (SHR-A1811, TH plus pyrotinib). **b** ROC curves comparing the HER2-LADDER predictive performance of single-agent chemotherapy with that of dual-targeted therapy (THP) and that of dual-agent chemotherapy with that of single-targeted therapy (TCbH/PCbH). **c** Tertile-based stratification of HER2-LADDER scores defining Low, Medium, and High groups aligned with therapeutic strategies. **d** Comparative analysis of pathological complete response (pCR) rates across HER2-LADDER-defined groups and different anti-HER2 treatment regimens (TCbHP/PCbHP, THP, TCbH/PCbH, SHR-A1811, TH plus pyrotinib), with statistical analysis by the chi-square test and Bonferroni adjustment. **e** Summary schematic illustrating treatment planning informed by HER2-LADDER scores: HER2-LADDER-Low patients are appropriate for de-escalation (THP or TCbH/PCbH), HER2-LADDER-Medium patients benefit from standard dual-targeted therapy (TCbHP/PCbHP), and HER2-LADDER-High patients may require alternative options such as ADCs or TKIs

With respect to monoclonal antibody-based regimens, logistic regression analyses revealed that HER2-LADDER scores were significantly correlated with treatment responses in both dual- and single-targeted anti-HER2 regimens (Fig. 3a). Notably, the TCbHP/PCbHP treatment cohort, comprising patients from the model construction set (*N* = 276), temporal validation set (*N* = 82), and trial-based validation set (*N* = 85), demonstrated a strong association between HER2-LADDER scores and treatment response (OR 5.349, 95% CI 3.899–7.338; *P* = 2.620 × 10^−25^). Specifically, significant associations were observed for dual HER2 blockade with taxane monochemotherapy (THP) (OR 5.224, 95% CI 1.597 − 17.084; *P* = 0.006) and for single-targeted TCbH/PCbH regimens (OR 2.623, 95% CI 2.033 − 3.385; *P* = 1.177 × 10^−13^). Consistent discrimination was observed across these groups, with an AUC = 0.842 for THP and an AUC = 0.785 for TCbH/PCbH (Fig. 3b). These results indicate that HER2-LADDER consistently predicts treatment response across monoclonal antibody-based anti-HER2 regimens, with notably stable performance in the de-escalated THP regimen.

To explore whether HER2-LADDER retains predictive relevance beyond monoclonal antibody-based regimens, we examined its association with treatment outcomes in cohorts receiving alternative anti-HER2 therapies. In the SHR-A1811 (novel anti-HER2 ADC) cohort, HER2-LADDER expression was moderately but significantly correlated with treatment outcomes (OR = 1.741, 95% CI 1.175–2.579; *P* = 0.006). In contrast, no significant association was detected in the TH plus pyrotinib cohort (OR = 1.107, 95% CI 0.549–2.234; *P* = 0.776). These exploratory findings suggest that while the model retains partial predictive relevance in ADC-treated tumors, its applicability does not extend to TKI-based therapies. This discrepancy likely reflects intrinsic biological and mechanistic differences between ADCs and small-molecule TKIs relative to standard monoclonal antibody-based regimens.

To explore whether HER2-LADDER scores could facilitate response interpretation across different anti-HER2 regimens, we stratified patients into three groups on the basis of score distribution within the model construction cohort. Using a tertile-based division, the entire score range was objectively partitioned into Low, Medium, and High groups. This segmentation ensured reproducibility and generalizability, providing a consistent framework for exploratory comparisons of treatment outcomes across different regimens (Fig. 3c). Comparisons of the pCR rates across these groups revealed distinct treatment response patterns (Fig. 3d). In the HER2-LADDER-Low group, the pCR rates were similarly high for TCbHP/PCbHP (96.2%), THP (85.7%, Bonferroni-adjusted *P* = 1 vs. TCbHP/PCbHP), and TCbH/PCbH (84.9%, *P* = 0.143), indicating potential appropriateness for regimen de-escalation. In the HER2-LADDER-Medium group, TCbHP/PCbHP significantly outperformed THP (pCR 80.0% vs. 7.7%, *P* = 6.382 × 10^−7^) and TCbH/PCbH (47.1%, *P* = 6.856 × 10^−5^), supporting maintenance of the standard regimen. In the HER2-LADDER-High group, patients who received SHR-A1811 (54.2%, *P* = 6.914 × 10^−3^) and TH plus pyrotinib (50.0%, *P* = 0.085) had poor responses to TCbHP/PCbHP (19.6%) but relatively better outcomes.

Collectively, these observations suggest that HER2-LADDER scores may provide a framework to assist in stratified therapeutic planning, supporting potential de-escalation regimens (THP, TCbH/PCbH) for HER2-LADDER-Low patients, standard-of-care TCbHP/PCbHP in HER2-LADDER-Medium patients, and alternative regimens (ADCs or TKIs) for HER2-LADDER-High patients (Fig. 3e). Together, HER2-LADDER represents a conceptually innovative and clinically translatable framework for precision-guided optimization of anti-HER2 therapies.

### Prognostic value of HER2-LADDER in the adjuvant treatment setting

**Fig. 4:**
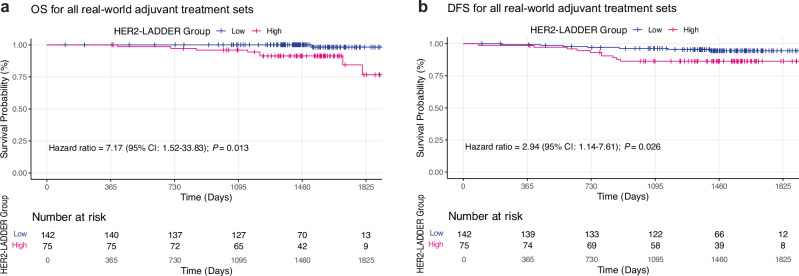
Prediction performance of the HER2-LADDER model in adjuvant-treated HER2-positive breast cancer. **a** Kaplan–Meier analysis comparing overall survival (OS) between the HER2-LADDER-High and HER2-LADDER-Low groups; hazard ratios and *P* value derived from the Cox proportional hazards model and log-rank test. **b** Kaplan‒Meier curve for disease-free survival (DFS) comparing the HER2-LADDER-High and HER2-LADDER-Low groups; hazard ratios and *P* value determined by Cox regression and log-rank tests. The extended subgroup analysis is provided in Supplementary Fig. 5

Given the central role of dual HER2-targeted therapy combined with chemotherapy in the adjuvant management of early-stage HER2-positive breast cancer, we further evaluated the prognostic utility of the HER2-LADDER model beyond the neoadjuvant context. Stratification on the basis of baseline HER2-LADDER scores revealed marked differences in long-term clinical outcomes. Patients in the High group had significantly worse overall survival (OS; hazard ratio (HR) 7.17; 95% CI: 1.52–33.83; log-rank *P* = 0.013; Fig. 4a) and worse DFS (HR 2.94; 95% CI: 1.14–7.61; *P* = 0.026; Fig. 4b) than those in the HER2-LADDER-Low group did. Notably, subgroup analysis of patients receiving adjuvant TCbHP further confirmed the prognostic relevance of HER2-LADDER, with the High group showing significantly inferior DFS (HR 4.71, 95% CI: 1.21–24.28; *P* = 0.024; Supplementary Fig. 5). These results indicate that HER2-LADDER scores, derived solely from pretreatment core-needle biopsy slides, can serve as powerful prognostic indicators to predict long-term outcomes, even in the adjuvant setting.

### Biological interpretability of the predictive features

**Fig. 5:**
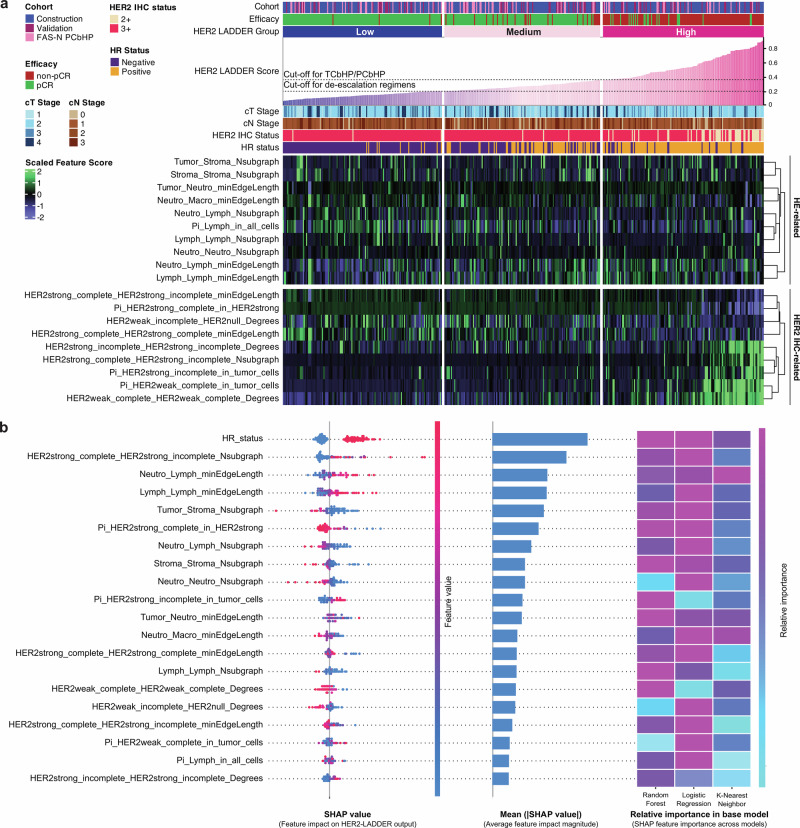
Interpretability deconvolution of the HER2-LADDER model. **a** Categorization of the key predictive spatial variables integrated into HER2-LADDER, derived from H&E (spatial interactions of tumor microenvironmental cells) and HER2 IHC (spatial interactions of HER2-expressing tumor cells). Variables include cell proportions (Pi), aggregation scale (Nsubgraph), edge-length metrics (minEdgeLength), and spatial centrality (Degrees). **b** Shapley additive explanations (SHAP) analysis quantifying the relative contributions of each feature and clinicopathological variable within the submodels and the final ensemble HER2-LADDER model. Features ranked by predictive importance (mean [|SHAP value|]), reflecting their overall importance

Building on the strong predictive performance of HER2-LADDER in both neoadjuvant and adjuvant settings, we next explored the biological basis of the key features of the model to better understand the mechanisms underlying the response to TCbHP/PCbHP treatment. The final feature set, derived through systematic engineering, integrated clinicopathological variables (e.g., HR status) with spatially resolved features extracted from digital pathology images. These spatial features were categorized according to staining modality and biological relevance (Fig. 5a).

From H&E-stained images, selected variables primarily reflected tumor microenvironment interactions, including the spatial distribution and colocalization patterns of lymphocytes, neutrophils, macrophages, and stromal cells relative to tumor cells. Representative features included Neutro_Lymph_Nsubgraph (cellular aggregation), Lymph_Lymph_Nsubgraph (cellular aggregation), Pi_Lymph_in_all_cells (proportion), and Neutro_Lymph_minEdgeLength (intercellular distance). Moreover, features derived from HER2 IHC-stained images described the spatial organization and expression heterogeneity of HER2-positive tumor cells, encompassing the proportions of HER2-expressing cell subtypes and their spatial arrangements. Representative variables included HER2strong_complete_HER2strong_incomplete_Nsubgraph (cellular aggregation), Pi_HER2strong_complete_in_HER2strong (proportion), Pi_HER2strong_incomplete_in_tumor_cells (proportion), and HER2strong_complete_HER2strong_complete_minEdgeLength (intercellular distance).

To assess the relative contribution of these features, we applied Shapley additive explanations (SHAP) analysis across individual submodels and the ensemble voting classifier (Fig. 5b). SHAP confirmed that both H&E-derived microenvironmental cell interaction features and HER2 IHC-derived tumor cell spatial characteristics substantially influenced the model predictions. These findings underscore that the most influential features identified by HER2-LADDER are not only predictive but also biologically meaningful, offering insights into the spatial determinants of therapeutic response.

### Spatial determinants of treatment responsiveness suggested by the HER2-LADDER model

**Fig. 6:**
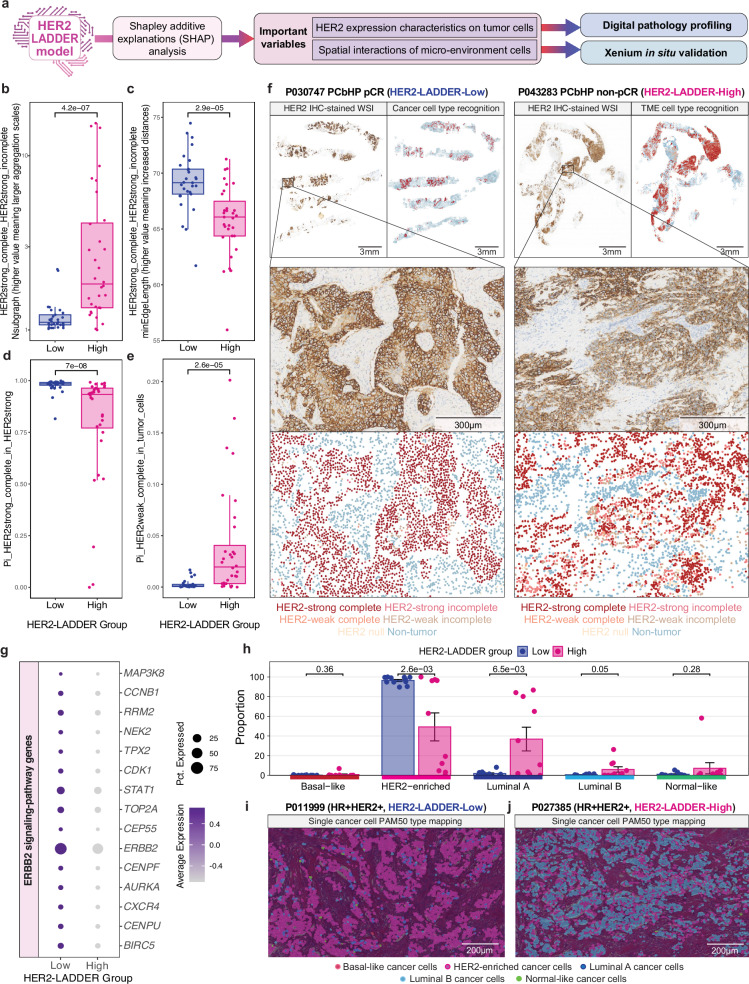
Spatial multiomics characterization of HER2 expression heterogeneity determinants in HER2-LADDER groups. **a** Analytical framework illustrating the integration of digital pathology profiling and Xenium in situ validation of HER2-expressing tumor cell spatial characteristics. **b** **c** Comparative analysis of the aggregation scale (**b**, HER2strong_complete_HER2strong_incomplete_Nsubgraph) and minimum intercellular distance (**c**, HER2strong_complete_HER2strong_incomplete_minEdgeLength) between HER2-strong complete and HER2-strong incomplete cells across HER2-LADDER groups; statistical significance was assessed by Wilcoxon tests. **d**, **e** Comparison of the proportions of HER2-strong complete (**d**) and HER2-weak complete (**e**) cells between the HER2-LADDER groups; statistical significance was assessed by Wilcoxon tests. **f** Representative HER2 IHC whole-slide images and tumor cell-type mapping illustrating spatial heterogeneity in HER2 membrane expression patterns between the HER2-LADDER groups. **g** Differential gene expression analysis from Xenium spatial transcriptomics (21 samples; 11 HER2-LADDER-Low and 10 HER2-LADDER-High), highlighting increased ERBB2 pathway gene expression in HER2-LADDER-Low tumors. **h** Single-cell PAM50 subtyping demonstrating significantly higher proportions of the HER2-enriched subtype in HER2-LADDER-Low tumors than in luminal subtypes in HER2-LADDER-High tumors; statistical significance was assessed by the Wilcoxon test. **i**–**j** Spatially resolved single-cell mappings of PAM50 subtypes showing distinct spatial distribution patterns for unified HER2-enriched cells (**i**, HER2-LADDER-Low tumors) versus mixed luminal and HER2-enriched patterns (**j**, HER2-LADDER-High tumors)

To further elucidate the biological mechanisms underlying the predictive ability of HER2-LADDER, we systematically investigated spatial determinants associated with treatment responsiveness, categorizing them into HER2 expression characteristics and tumor immune microenvironment features, as outlined in Fig. 6a. By integrating digital pathology profiling with spatial transcriptomics validation via Xenium in situ analyses, we closely examined key variables identified through SHAP analyses.

In terms of HER2 expression characteristics, marked spatial differences emerged between tumors classified into the HER2-LADDER-High group and those classified into the HER2-LADDER-Low group. Specifically, HER2-LADDER-High tumors exhibited significantly greater heterogeneity in HER2 membrane staining, characterized by mosaic-like distribution patterns. The average aggregation scale (HER2strong_complete_HER2strong_incomplete_Nsubgraph) was significantly greater in HER2-LADDER-High tumors (Wilcoxon *P* = 4.2 × 10^−7^; Fig. 6b). Additionally, these tumors displayed notably shorter average minimal intercellular distances between HER2-strong complete and incomplete membrane-expressing tumor cells (HER2strong_complete_HER2strong_incomplete_minEdgeLength; *P* = 2.9 × 10^−5^; Fig. 6c). Quantitative analysis further revealed that HER2-LADDER-High tumors contained significantly lower proportions of tumor cells with complete membrane HER2-strong positivity (Pi_HER2strong_complete_in_HER2strong; *P* = 7 × 10^−8^; Fig. 6d) and higher proportions of tumor cells with HER2-weak complete membrane staining (Pi_HER2weak_complete_in_tumor_cells; *P* = 2.6 × 10^−5^; Fig. 6e). These findings were visually confirmed via HER2 IHC WSIs, underscoring the pronounced mosaic pattern of HER2 expression as a potential determinant of reduced sensitivity to dual HER2-targeted treatments (Fig. 6f).

Complementary Xenium in situ analysis validated these observations at the transcriptomic level, demonstrating significantly elevated expression of ERBB2 signaling-related genes and downstream effectors in tumors from the HER2-LADDER-Low group, indicating robust HER2 pathway activation (Fig. 6g). Single-cell PAM50 subtyping supported these findings, as HER2-LADDER-Low tumors exhibited a significantly greater proportion of HER2-enriched subtype cells (*P* = 2.6 × 10^−3^), whereas HER2-LADDER-High tumors had greater proportions of luminal A (*P* = 6.5 × 10^−3^) and luminal B subtype cells (*P* = 0.05; Fig. 6h). Spatial mapping further confirmed a relatively unified composition of HER2-enriched cells within HER2-LADDER-Low tumors (Fig. 6i), which contrasted sharply with the mosaic-like distribution observed in HER2-LADDER-High tumors dominated by cells of luminal subtypes (Fig. 6j).

**Fig. 7:**
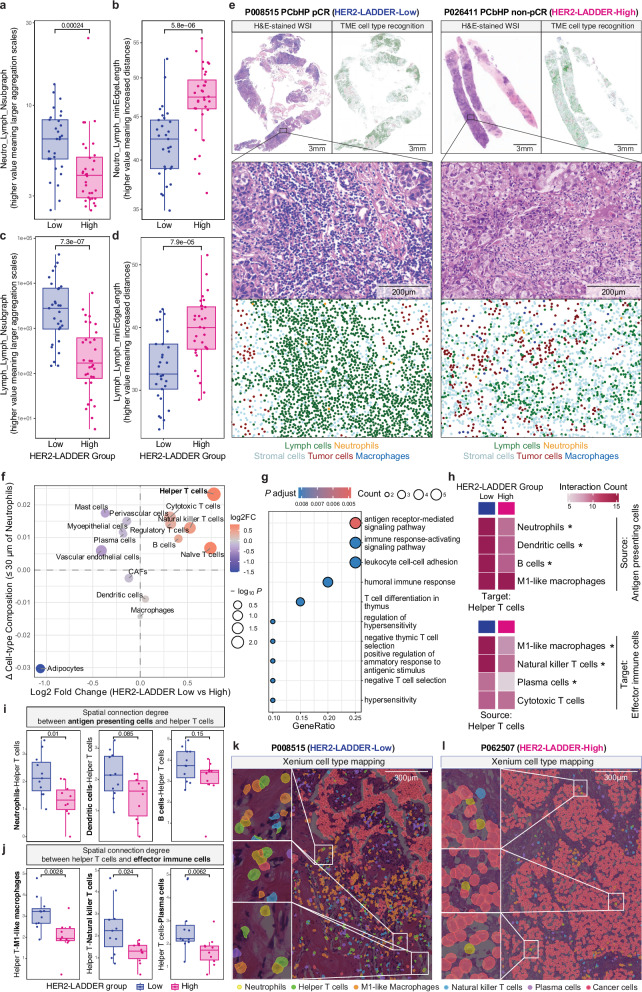
Spatial multiomics characterization of immune microenvironment determinants associated with HER2–LADDER groups. **a**, **b** Comparative analyses of neutrophil–lymphocyte aggregation scales (**a**, Neutro_Lymph_Nsubgraph) and minimum intercellular distances (**b**, Neutro_Lymph_minEdgeLength) between HER2-LADDER groups; statistical significance was assessed by Wilcoxon tests. **c**, **d** Aggregation scales (**c**, Lymph_Lymph_Nsubgraph) and minimum intercellular distances (**d**, Lymph_Lymph_minEdgeLength) within lymphocyte populations compared with HER2-LADDER groups; statistical significance was assessed by Wilcoxon tests. **e** Representative spatial mappings from H&E WSIs depicting differential immune cell clustering patterns (neutrophil‒lymphocyte and lymphocyte‒lymphocyte interactions) between the HER2-LADDER groups. **f** Comparative Xenium-derived cell composition analysis, highlighting significantly increased helper T-cell proportions in HER2-LADDER-Low tumors, particularly within 30 μm of neutrophils; statistical significance was assessed by Wilcoxon tests. **g** Pathway enrichment analysis of differentially expressed genes in neutrophils derived from Xenium spatial transcriptomic profiling of 21 tumor samples (11 HER2-LADDER-Low and 10 HER2-LADDER-High cases). **h** Cell–cell ligand–receptor interaction analysis identifying significant differences in interactions between antigen-presenting cells (neutrophils, dendritic cells, and B cells) and helper T cells, as well as subsequent interactions between helper T cells and effector immune cells (M1-like macrophages, natural killer T cells, and plasma cells), between the HER2-LADDER groups; statistical significance is indicated. **i**, **l** Spatial connectivity analyses using sc-MTOP quantitatively confirm stronger interactions in the HER2-LADDER-Low groups between neutrophils and helper T cells (**i**) and subsequently between helper T cells and effector immune populations (**j**), suggesting that coordinated immune responses drive enhanced treatment sensitivity. Statistical significance was assessed by the Wilcoxon test. Representative spatial interaction schematics are illustrated in (**k**, **l**)

When the tumor immune microenvironment was examined, significant differences in spatial immune cell organization were observed. Tumors with better therapeutic responses demonstrated characteristics consistent with an “immune-hot” phenotype, including increased aggregation scales (Neutro_Lymph_Nsubgraph; *P* = 2.4 × 10^−4^; Fig. 7a) and reduced minimal intercellular distances (Neutro_Lymph_minEdgeLength; *P* = 5.8 × 10^−6^; Fig. 7b) between neutrophils and lymphocytes. Similar immune-hot patterns were observed for lymphocyte‒lymphocyte interactions, including larger aggregation scales (Lymph_Lymph_Nsubgraph; *P* = 7.3 × 10^−7^; Fig. 7c) and shorter intercellular distances (Lymph_Lymph_minEdgeLength; *P* = 7.9 × 10^−5^; Fig. 7d). Spatial mappings from H&E-stained WSIs visually validated these findings, highlighting dense immune cell clustering within tumors exhibiting favorable responses (Fig. 7e).

Building upon these observations, the results of the Xenium analysis further revealed that the abundance of helper T cells differed most markedly between the HER2-LADDER-Low and HER2-LADDER-High groups (*P* = 3.8 × 10^−3^; Fig. 7f). Further spatial analysis revealed an enrichment of helper T cells within 30 μm of neutrophils in HER2-LADDER-Low tumors, suggesting critical spatially mediated immunological interactions (Fig. 7f). In HER2-LADDER-Low tumors, differential expression analysis revealed significant enrichment of antigen receptor-mediated signaling pathways in neutrophils (Fig. 7g). These findings suggest that neutrophils may engage in antigen presentation, prompting further examination of their interactions with antigen-presenting cells (APCs).

Ligand‒receptor analyses subsequently revealed significant differences in cellular communication between APC populations (neutrophils, dendritic cells, and B cells) and helper T cells, as well as downstream interactions between helper T cells and effector immune populations, including M1-like macrophages, natural killer T cells, and plasma cells (Fig. 7h). Quantitative spatial interaction assessments indicated notably stronger connections between neutrophils and helper T cells (Wilcoxon *P* = 0.01; Fig. 7i), with subsequent robust interactions extending from helper T cells to effector immune cells (M1-like macrophages: *P* = 2.8 × 10^−3^; natural killer T cells: *P* = 0.024; plasma cells: *P* = 6.2 × 10^−3^; Fig. 7j). Collectively, these findings suggest that a spatial immunological cascade initiated by neutrophil-helper T-cell interactions enhances effector immune responses, thereby potentiating antitumor immunity and improving therapeutic responses (Fig. 7k, l).

In summary, the integration of digital pathology profiling with Xenium spatial in situ analyses provided mechanistic insights into the distinct spatial characteristics of HER2 expression and tumor microenvironmental interactions that underpin HER2-LADDER predictions. These findings reinforce the biological interpretability of HER2-LADDER, highlighting the pivotal role of spatially resolved tumor phenotypes in predicting therapeutic responsiveness.

## Discussion

In this study, we introduced HER2-LADDER, a spatially interpretable artificial intelligence framework that predicts individual responses to standard-of-care neoadjuvant dual HER2-targeted therapy in HER2-positive breast cancer.^14^ Unlike prior predictive models, which are primarily based on molecular or imaging biomarkers, HER2-LADDER uniquely integrates routine clinicopathological information with spatially resolved digital pathological features derived from standard core-needle biopsy H&E and HER2 IHC WSIs. Our strategy bridges the gap between computational pathology and spatial tumor biology, offering a precise, highly interpretable, and clinically accessible approach for individualized HER2-targeted therapeutic stratification.

Current clinical management of HER2-positive breast cancer employs dual HER2-targeted therapy combining trastuzumab and pertuzumab with chemotherapy as the neoadjuvant standard-of-care, achieving considerable success in improving pCR rates and survival outcomes.^10,34,35^ However, treatment responses vary considerably, underscoring the need for more tailored therapeutic strategies. Recent advances in HER2-positive breast cancer treatment have introduced several strategies. For example, genomic assays^24,36–38^ have emerged as valuable tools capable of predicting pCR and survival outcomes. Similarly, functional imaging approaches, such as 18F-FDG-PET,^17^ can effectively select patients suitable for chemotherapy de-escalation. However, these methods share common limitations: they are costly and resource-intensive and have limited availability in routine clinical practice. Additionally, multimodal deep-learning frameworks integrating MRI, WSIs, and clinical data have improved early pCR prediction accuracy across breast cancer subtypes.^39,40^ However, their specificity and precision for HER2-positive breast cancer remain suboptimal, as they typically operate as “black-box” models with limited interpretability, offering insufficient biological insight and lacking specific tailoring toward anti-HER2 targeted therapies, thereby limiting their clinical application.

HER2-LADDER addresses these limitations by leveraging routinely acquired digital pathology data and established clinical metrics, enhancing clinical accessibility and potential for widespread implementation. Its robust predictive capacity was demonstrated through rigorous validation in both real-world and trial-based cohorts, underscoring its reliability and clinical utility. Notably, HER2-LADDER effectively delineates distinct patient subgroups on the basis of predicted therapeutic response, enabling the use of tailored clinical strategies such as chemotherapy de-escalation for HER2-LADDER-Low patients and identifying those with limited benefit from conventional dual HER2 blockade who may warrant treatment alteration (e.g., next-generation ADCs or TKIs).

Clinically, the concept of therapeutic de-escalation has garnered significant attention, especially given the adverse effects and financial burdens associated with intensive treatment regimens.^41–43^ Our findings further support the ability of HER2-LADDER to identify patients who may safely undergo chemotherapy de-escalation or single-targeted anti-HER2 therapy without compromising clinical outcomes, while highlighting that HER2-LADDER-High patients exhibit suboptimal responses to standard TCbHP/PCbHP treatment and relatively better outcomes with next-generation ADCs or TKI-based regimens. Importantly, the modest predictive capability for ADCs and TKIs suggests fundamental mechanistic differences between these alternative agents and standard dual HER2-targeted monoclonal antibodies.^14,44^ These differences emphasize the need for tailored predictive models or biomarkers when therapeutic switching is considered.^45^

A major strength of HER2-LADDER lies in its robust biological interpretability. Spatial digital pathology profiling revealed HER2 expression heterogeneity as a critical determinant of therapeutic response.^45,46^ HER2-LADDER-High tumors demonstrated a distinct mosaic HER2 expression pattern, with intermixed clusters of tumor cells exhibiting strong, weak, and incomplete membrane staining. This spatial mosaicism has been increasingly recognized as a hallmark of intratumoral HER2 heterogeneity, which may limit the efficacy of conventional HER2-targeted antibodies reliant on uniform receptor expression for therapeutic engagement.^47^ Such heterogeneity may render tumors less responsive to trastuzumab and pertuzumab, which require adequate and complete HER2 membrane staining for optimal binding. In contrast, next-generation ADCs such as trastuzumab deruxtecan (T-DXd) or SHR-A1811 demonstrate enhanced efficacy in heterogeneously expressing tumors because of their ability to elicit bystander killing effects and payload diffusion across adjacent tumor cells regardless of the HER2 expression level.^48,49^ In addition, responses to TKIs may be less influenced by cell membrane heterogeneity and more dependent on downstream pathway activation.^44,50^ Therefore, our findings indicate that patients with HER2-LADDER-High tumors may benefit more from novel ADCs or TKIs than from conventional dual-targeted regimens.

In addition to tumor-intrinsic heterogeneity, spatial features of the immune microenvironment have emerged as pivotal predictors of treatment response.^51,52^ HER2-LADDER-Low tumors are characterized by dense and spatially coordinated clusters of lymphocytes and neutrophils. These spatial immune niches resemble inflamed tumor microenvironments known to facilitate improved responses to both chemotherapy and HER2-targeted agents. Among immune players, helper T cells were particularly enriched in proximity to neutrophils within responsive tumors, suggesting potential intercellular crosstalk. Xenium-based spatial transcriptomics and pathway enrichment analyses further revealed that neutrophils in these tumors upregulate antigen receptor-mediated signaling, suggesting that neutrophils are unconventional antigen-presenting cells (APCs) capable of activating T cells. This notion is supported by emerging studies showing that tumor-infiltrating neutrophils, under specific microenvironmental conditions, can acquire APC-like phenotypes and orchestrate adaptive immune responses.^53–56^ Together, these spatial and functional immune signatures reinforce a model in which neutrophil-helper T-cell interactions facilitate coordinated immune activation, contributing to superior antitumor immunity and an enhanced therapeutic response.

Despite comprehensive retrospective validation across multiple cohorts, this study has inherent limitations. First, the retrospective nature of the analysis, while informative, may be subject to selection biases and does not fully capture real-world implementation challenges. Second, the variability in staining protocols and digital image scanning methods across clinical centers could influence feature extraction consistency, and broader deployment requires ongoing efforts toward standardization. Prospective validation in independent cohorts, including within the ongoing FASCINATE-N trial (NCT05582499), will be crucial to definitively establish the clinical utility and impact of HER2-LADDER-guided therapeutic decision-making.

In conclusion, HER2-LADDER represents an advancement toward precision medicine in HER2-positive breast cancer. By linking spatial tumor architecture to therapeutic outcomes, HER2-LADDER provides clinically actionable predictions to optimize therapeutic strategies in diverse clinical settings. Future studies involving larger, prospective, and geographically diverse populations will further refine the model’s predictive capacity and enhance its clinical applicability, ultimately contributing to improved therapeutic outcomes and patient care.

## Materials and methods

### Clinical cohorts and study design

This study integrated both real-world treatment cohorts from Fudan University Shanghai Cancer Center (FUSCC) and clinical trial patients to support model development, validation, and clinical translation. For model development, a total of 372 treatment-naïve patients who completed neoadjuvant dual HER2-targeted therapy with six cycles of standard taxane, carboplatin, trastuzumab, and pertuzumab (TCbHP/PCbHP) between 2020 and 2024 at FUSCC were screened. After 14 patients without paired baseline H&E and HER2 IHC slides were excluded, 358 patients were eligible for spatial computational pathology analysis. Among them, 276 patients treated between 2020 and 2022 constituted the model construction set, while 82 patients treated after 2022 formed a temporal validation set to evaluate model reproducibility in more recent real-world cases, which is a validation strategy that guards against overfitting, mitigates temporal drift in data distributions, and more faithfully estimates the model’s prospective performance.^57^ pCR was determined after surgery on the basis of standard definitions and was confirmed by two independent pathologists.

To further assess cross-center generalizability, an independent external validation cohort comprising 40 HER2-positive breast cancer patients treated with neoadjuvant TCbHP between 2020 and 2025 was constructed at Chongqing University Cancer Hospital (CQUCH). For all patients across cohorts, paired baseline core-needle biopsy H&E and HER2 IHC WSIs were available and scanned at 40 × magnification. All the images were processed through the same feature extraction pipeline, and the pretrained HER2-LADDER model was applied to the external cohort without retraining or parameter adjustment.

The FASCINATE-N (NCT05582499) prospective, single-arm, phase II clinical trial was conducted at FUSCC between December 27, 2022, and February 11, 2024. A total of 88 treatment-naïve women with HER2-positive breast cancer were enrolled and received six cycles of neoadjuvant therapy consisting of nab-paclitaxel, carboplatin, trastuzumab, and pertuzumab (PCbHP). All patients underwent core-needle biopsies prior to treatment, and formalin-fixed, paraffin-embedded (FFPE) sections were subjected to hematoxylin and eosin (H&E) staining and HER2 immunohistochemistry (IHC), followed by digital whole-slide scanning for spatial computational pathology analysis. Among them, 85 patients had paired high-quality H&E and HER2 IHC WSIs available and were included as a trial-based validation cohort.

For external validation, several independent retrospective cohorts from FUSCC were included. The neoadjuvant validation cohort (*N* = 490) comprised HER2-positive patients treated with various anti-HER2–based regimens, including THP, TCbH/PCbH, SHR-A1811, and TH plus pyrotinib, with corresponding paired pretreatment H&E and HER2 IHC WSIs. The adjuvant validation cohort (*N* = 282) included HER2-positive patients who had completed curative-intent surgery and adjuvant dual HER2-targeted therapy, with long-term follow-up data for disease-free survival (DFS) and overall survival (OS). HER2 status was determined according to the ASCO/CAP 2023 guidelines,^58^ and all clinicopathological data and treatment outcomes were reviewed and verified by board-certified oncologists and pathologists. All slides were processed through a standardized digital pathology workflow to ensure reproducible spatial feature extraction across cohorts. Written informed consent was obtained from all participants in accordance with the institutional ethical guidelines of Fudan University Shanghai Cancer Center (approval number: 050432-4-2108).

### Whole-slide image processing

Baseline core-needle biopsies were FFPE, sectioned, and subjected to H&E and HER2 IHC staining. WSIs were digitally scanned at 40× magnification using the same slide scanner (NanoZoomer, Hamamatsu) across all cohorts to ensure consistency of image resolution and color fidelity. Image quality was uniformly assessed by two experienced pathologists, ensuring adequate tissue representation, sharp nuclear and membranous staining, and the absence of scanning or staining artifacts. Patients with incomplete tumor regions, tissue folds, or out-of-focus areas were excluded. To harmonize data quality across cohorts from different time periods and clinical settings, a standardized image preprocessing pipeline was applied before computational feature extraction, including color normalization, background correction, and artifact removal. This ensured comparable input quality for downstream spatial and morphological analyses regardless of cohort origin.

### Computational pathology feature extraction

To achieve precise digital profiling of tumor and microenvironmental cells, we employed two deep learning–based cell segmentation and classification algorithms, using HoVer-Net for H&E-stained images and D-PathAI for HER2 IHC-stained images, with customized enhancements for HER2-specific membrane pattern analysis.

For H&E-stained WSIs, cell segmentation and classification were performed using HoVer-Net, a multitask convolutional neural network architecture designed to jointly perform nuclear segmentation and cell-type classification.^59^ HoVer-Net simultaneously learns horizontal and vertical (HoVer) gradients of nuclei to resolve overlapping or clustered nuclei and uses an auxiliary classification branch to assign cell identity. In this study, we used the official HoVer-Net pretrained model released by the original authors, which was trained and validated on large, expert-annotated public datasets, including PanNuke^60^ and MoNuSAC.^61^ These datasets provide extensive coverage of nuclear morphologies and cell-type annotations, forming the established training and validation foundation of the model, and enabling robust identification of the five major cell types considered in this study (tumor cells, lymphocytes, neutrophils, stromal cells, and macrophages). Only high-confidence segmented nuclei were retained for downstream analysis. Nuclear instances were obtained exclusively from the standard HoVer-Net postprocessing pipeline, and no additional confidence scoring, manual filtering, or newly introduced quality control algorithms were applied. Consistent with the HoVer-Net architecture and prior sc-MTOP applications, nuclei with incomplete or unreliable predictions near patch boundaries were inherently excluded.

For HER2 IHC-stained WSIs, we used a customized pipeline based on D-PathAI, a CNN tailored for IHC analysis of membranous protein expression.^62^ D-PathAI performs pixelwise classification to segment tumor cells and then extracts membrane contours for evaluating both HER2 staining intensity and membrane completeness, in line with clinical HER2 scoring guidelines from ASCO/CAP.^58^ Specifically, tumor cells were assigned to five categories on the basis of complete or incomplete membranous staining and staining intensity: HER2strong_complete, HER2strong_incomplete, HER2weak_complete, HER2weak_incomplete, and HER2_null. To achieve this goal, the pipeline integrated a two-branch deep learning model: one branch for membrane integrity detection using edge enhancement and contour-based postprocessing and a second for staining intensity classification using pixel distribution and DAB color deconvolution. The result was a high-resolution tumor map that preserved both the spatial localization and heterogeneity of HER2-positive subpopulations, allowing downstream quantification of spatial colocalization, edge connectivity, and subgraph interactions among HER2-expressing tumor cells.

All extracted cell coordinates and identities were passed into the sc-MTOP framework^31^ to derive spatial metrics, including intercellular edge distances, subgraph clustering (Nsubgraph), spatial proximity indices, and local centrality measures (node degrees). These metrics enabled the quantification of both tumor architecture and immune cell–tumor interactions, forming the spatial basis of the predictive features of HER2-LADDER.

### Construction of the HER2-LADDER prediction framework

The HER2-LADDER predictive framework was developed to stratify patient response to standard dual HER2-targeted neoadjuvant therapy. Model development was performed exclusively in Python (version 3.12), integrating structured clinicopathological data and high-dimensional digital pathology features extracted from pretreatment H&E and HER2 IHC WSIs.

An initial pool of 143 candidate variables was curated. The four clinicopathological variables included age, clinical T stage, clinical N stage, and HR status, and 139 quantitative pathological features capturing cellular composition, spatial architecture, HER2 membrane expression heterogeneity, and tumor microenvironmental cell interactions. To prevent multicollinearity and reduce overfitting, a stepwise feature reduction pipeline was implemented. First, Pearson correlation analysis was performed to eliminate variables with high collinearity (|*r*|>0.75). Next, univariate association tests (Wilcoxon rank-sum) between each feature and pCR outcome were conducted to remove weakly discriminatory variables. A final subset of 20 robust features with high discriminative capacity was retained for model construction.

Three supervised learning algorithms were selected for their complementary strengths in handling nonlinearities, sparsity, and multivariate interactions: random forest for robust variable selection and interaction modeling; logistic regression with L2 regularization for probabilistic interpretability; and K-nearest neighbors (KNN) for local structure-sensitive classification. Each algorithm underwent fivefold cross-validation and grid-based hyperparameter optimization using the GridSearchCV module from scikit-learn. Hyperparameter optimization for each base learner was performed exclusively within the Model Construction Set using fivefold stratified cross-validation (StratifiedKFold) by treatment efficacy. The area under the receiver operating characteristic curve (AUC-ROC) was used as the optimization metric. A grid-based hyperparameter search was implemented using the GridSearchCV module from scikit-learn. All preprocessing steps were performed within each training fold to avoid information leakage.

For the random forest classifier, all parameter combinations were tested across n_estimators from 50 to 200 (step size 50), max_depths including None, 5, 10, and 20, and min_samples_split set to 2, 5, and 10. For logistic regression, the hyperparameter space included regularization strengths (C) of 0.01 to 100 (log scale) and iteration limits (max_iter) from 50 to 500. For KNN, the number of neighbors ranged from 3 to 9, and n_jobs was tuned from None to 20 to parallelize the computations. The optimal combination for each classifier was selected on the basis of the AUC performance in fivefold cross-validation. The three optimized classifiers were integrated via a soft voting ensemble strategy, yielding a final prediction score for each patient, representing the estimated probability of non-pCR under standard TCbHP/PCbHP treatment.

To derive a binary classification for downstream analyses, the optimal decision threshold was determined by maximizing the Youden index (sensitivity + specificity − 1) using patient-level predicted probabilities from the Model Construction Set. This threshold was fixed and subsequently applied to the temporal validation set and external validation sets. Separately, for clinically meaningful and reproducible risk stratification, HER2-LADDER scores were further categorized into Low, Medium, and High groups on the basis of the tertile distribution within the Model Construction Set. Specifically, patients were classified into HER2-LADDER-Low, -Medium, and -High groups according to the lower and upper tertiles of the score distribution. This stratification ensures objective subgroup delineation independent of outcome labels and facilitates external generalizability. The tertile-based division, widely adopted in comparable prognostic modeling studies,^63^ provides a statistically transparent and reproducible framework for clinical implementation. Collectively, this hierarchical stratification links model-derived prediction scores with therapeutic regimens, thereby enhancing the translational utility of HER2-LADDER in guiding individualized HER2-targeted treatment strategies.

### Performance evaluation metrics

The predictive performance of HER2-LADDER was comprehensively evaluated using multiple statistical and clinical utility metrics. Model discrimination was primarily assessed by calculating the area under the receiver operating characteristic curve (AUC) and average precision (AP) for both the model construction set and the external validation cohorts. Pairwise AUC comparisons were conducted using the DeLong test to assess statistical significance. These metrics were computed using the scikit-learn library to reflect the model’s sensitivity-specificity tradeoff and precision-recall performance.

To assess prediction calibration, classification consistency, and agreement between the predicted and observed labels, we carried out confusion matrix analyses, from which the accuracy, sensitivity, specificity, positive predictive value, and negative predictive value were obtained. We also computed Cohen’s kappa statistic as a measure of classification agreement beyond chance, which is especially important given imbalanced outcome distributions. Calibration curves were plotted to compare predicted probabilities against observed response rates across deciles or risk bins, and model calibration was quantified using the Brier score. A lower Brier score indicates better probabilistic calibration and reliability of risk estimates.

Decision curve analysis (DCA) was conducted using the pyDCA package to evaluate the net clinical benefit of HER2-LADDER across a spectrum of probability thresholds, quantifying the relative utility of model-based decisions compared to “treat-all” or “treat-none” strategies. The DCA results demonstrated that HER2-LADDER achieved superior net benefit across clinically relevant threshold ranges. Finally, to evaluate robustness and generalizability, the final HER2-LADDER model was independently applied to external validation cohorts in both the neoadjuvant and the adjuvant settings. Discrimination performance was assessed using the AUC and AP as previously described. Classification agreement was further evaluated using confusion matrices, from which accuracy, sensitivity, specificity, and Cohen’s kappa were derived (Supplementary Fig. 2a–c, f). To examine the calibration, we generated calibration curves by grouping patients into deciles according to the predicted probabilities and plotting the observed event proportions against the average predicted probabilities in each bin (Supplementary Fig. 2d). In parallel, we computed the Brier score, defined as the mean squared difference between the predicted probabilities and actual outcomes. These calibration assessments complement discrimination metrics by quantifying how well predicted probabilities align with true response frequencies, thereby strengthening claims about the model’s clinical reliability.^64^

### Model interpretability

To enhance clinical trust and biological relevance, we employed a post hoc explainable AI framework based on SHapley Additive exPlanations (SHAP) to quantify the contribution of each feature to individual patient predictions.^65^ SHAP analysis was performed using the shap Python package and enabled both global feature importance ranking and local interpretability of predictions at the single-patient level. Global SHAP summary plots revealed that spatial variables related to HER2 membrane expression heterogeneity, such as HER2strong_complete_HER2strong_incomplete_Nsubgraph, and immune cell spatial coordination metrics, such as Neutro_Lymph_Nsubgraph, were influential features across all the submodels and the final ensemble. These findings highlight the dual importance of tumor-intrinsic heterogeneity and immune microenvironment topology in predicting the response to dual HER2-targeted therapy. At the local level, individual SHAP force plots were generated to visualize how specific spatial features influenced the prediction scores in each case.

### Xenium spatial transcriptomic profiling

To validate spatial features identified from computational pathology and elucidate underlying biological mechanisms associated with therapeutic response, in situ single-cell spatial transcriptomic profiling was performed on 21 FFPE tumor samples (11 HER2-LADDER-Low and 10 HER2-LADDER-High cases) using the Xenium platform (10x Genomics). Formalin-fixed, paraffin-embedded (FFPE) tumor tissues were selected from representative cases across HER2-LADDER score strata, with spatial profiling conducted on annotated regions encompassing tumor-enriched and immune-infiltrated areas. Each 5-μm section was processed following the manufacturer’s instructions.

A custom 380-gene panel targeting breast cancer–relevant genes, immune activation markers, stromal components, and HER2-related pathways was employed. Raw transcript counts were normalized and processed using the Xenium secondary analysis pipeline, and downstream single-cell analysis was conducted in Seurat (v5.3). Following layer integration and graph-based clustering (FindNeighbors, FindClusters), cells were annotated according to canonical markers derived from CellMarker 2.0. The broad cell classes included epithelial (malignant), stromal, myeloid, lymphoid, and endothelial populations. Epithelial tumor cells were further classified using PAM50-based subtype enrichment scoring and categorized as HER2-enriched, luminal A, luminal B, basal-like, or normal-like. Immune cell subtypes were refined using curated gene modules into helper T cells, cytotoxic T cells, natural killer T cells, plasma cells, M1/M2-like macrophages, dendritic cells, mast cells, and neutrophils.

To characterize the spatial organization of cell types, 2D coordinates from Xenium images were mapped onto single-cell identities. Spatial networks were constructed using the Giotto package based on Delaunay triangulation with a maximum distance criterion to define neighbor relations. For each cell, a local neighborhood matrix was computed using the composition of the nearest 200 cells; these matrices were integrated across tissues to define distinct spatial niches via Leiden clustering, following the approach described in a previously published study.^33^ The sc-MTOP framework was applied to quantify graph-theoretic topological features (e.g., edge length, degree, clustering coefficient, and betweenness centrality) between tumor cells and microenvironmental components.

Ligand‒receptor interaction analyses were conducted using the CellChat package to infer intercellular communication networks. Differences in signaling strength and pathway utilization were computed between HER2-LADDER-defined groups, highlighting distinct interaction axes—particularly between antigen-presenting cells (APCs) and helper T cells, as well as between helper T cells and downstream effector populations (e.g., M1-like macrophages, plasma cells, and natural killer T cells).

Differential gene expression analysis between spatially defined subpopulations was performed with Wilcoxon rank-sum tests, followed by Benjamini–Hochberg false discovery rate (FDR) correction. Genes significantly enriched in HER2-LADDER-Low tumors included ERBB2 pathway components, antigen presentation machinery, and immune effector markers, supporting the functional activation of antitumor immunity in spatially immune-hot niches. Overall, this integrative spatial transcriptomic analysis provided orthogonal molecular validation of spatial pathology-derived features and revealed distinct immune and oncogenic signaling architectures underlying the therapeutic response to dual HER2-targeted therapy.

### Statistical analysis

Continuous variables were summarized using means and standard deviations, with comparisons conducted via *t*-tests or Mann‒Whitney tests depending on the data distribution. Categorical variables were compared using Fisher’s exact test or chi-square tests. Logistic regression was employed for univariate and multivariate analyses, with odds ratios (ORs) and 95% confidence intervals (CIs) calculated. Survival analyses for the adjuvant cohort utilized Kaplan‒Meier curves and Cox proportional hazards regression models, with log-rank tests to compare groups. Spatial metric differences and molecular expression levels between patient groups were assessed using Wilcoxon rank-sum tests. A two-sided *P* value <0.05 was considered to indicate statistical significance. Analyses were conducted using R (version 4.4.3) and Python (version 3.12) software.

## Supplementary information

Supplementary Materials

## Data Availability

All engineered feature data used for model development and downstream analyses are publicly available in Supplementary Table 1. The Xenium spatial transcriptomics data generated in this study are available through the National Omics Data Encyclopedia (NODE) under accession number OEP00006015. The computational framework relies on publicly available algorithms, including HoVer-Net (https://github.com/vqdang/hover_net), the nuclei- and membrane-aware HER2 segmentation framework (https://github.com/Lion-shine/Segment-Membranes-and-Nuclei-from-Histopathological-Images-via-Nuclei-Point-level-Supervision), and sc-MTOP (https://github.com/fuscc-deep-path/sc_MTOP). The modeling workflow is described in detail in the Methods section and summarized in Supplementary Fig. 3. Owing to ethical approval constraints and patient privacy regulations, raw whole-slide images (WSIs) and full annotation data cannot be publicly released. Deidentified data and additional materials may be made available upon reasonable request to the corresponding author, subject to institutional approval and data use agreements.

## References

[1] Loibl, S. & Gianni, L. HER2-positive breast cancer. *Lancet* **389**, 2415–2429 (2017). 27939064 10.1016/S0140-6736(16)32417-5

[2] Wu, J. et al. CACA guidelines for holistic integrative management of breast cancer. *Holist. Integr. Oncol.* **1**, 7 (2022). 37520336 10.1007/s44178-022-00007-8 PMC9255514

[3] Slamon, D. J. et al. Human breast cancer: correlation of relapse and survival with amplification of the HER-2/neu oncogene. *Science* **235**, 177–182 (1987). 3798106 10.1126/science.3798106

[4] Swain, S. M., Shastry, M. & Hamilton, E. Targeting HER2-positive breast cancer: advances and future directions. *Nat. Rev. Drug Discov.* **22**, 101–126 (2023). 36344672 10.1038/s41573-022-00579-0 PMC9640784

[5] Gentile, G., Gerosa, R., de Azambuja, E. & Piccart-Gebhart, M. 20th anniversary of adjuvant trastuzumab: reflections on a breakthrough moment. *Ann. Oncol.* **37**, 470–480 (2025). 41386295 10.1016/j.annonc.2025.12.002

[6] Cameron, D. et al. 11 years’ follow-up of trastuzumab after adjuvant chemotherapy in HER2-positive early breast cancer: final analysis of the HERceptin adjuvant (HERA) trial. *Lancet* **389**, 1195–1205 (2017). 28215665 10.1016/S0140-6736(16)32616-2 PMC5465633

[7] Slamon, D. et al. Adjuvant trastuzumab in HER2-positive breast cancer. *N. Engl. J. Med.* **365**, 1273–1283 (2011). 21991949 10.1056/NEJMoa0910383 PMC3268553

[8] Gianni, L. et al. Neoadjuvant chemotherapy with trastuzumab followed by adjuvant trastuzumab versus neoadjuvant chemotherapy alone, in patients with HER2-positive locally advanced breast cancer (the NOAH trial): a randomised controlled superiority trial with a parallel HER2-negative cohort. *Lancet* **375**, 377–384 (2010). 20113825 10.1016/S0140-6736(09)61964-4

[9] Gianni, L. et al. Efficacy and safety of neoadjuvant pertuzumab and trastuzumab in women with locally advanced, inflammatory, or early HER2-positive breast cancer (NeoSphere): a randomised multicentre, open-label, phase 2 trial. *Lancet Oncol.* **13**, 25–32 (2012). 22153890 10.1016/S1470-2045(11)70336-9

[10] Shao, Z. et al. Efficacy, safety, and tolerability of pertuzumab, trastuzumab, and docetaxel for patients with early or locally advanced ERBB2-positive breast cancer in Asia: the PEONY phase 3 randomized clinical trial. *JAMA Oncol.* **6**, e193692 (2020). 31647503 10.1001/jamaoncol.2019.3692 PMC6813591

[11] Huang, L. et al. Neoadjuvant-adjuvant pertuzumab in HER2-positive early breast cancer: final analysis of the randomized phase III PEONY trial. *Nat. Commun.* **15**, 2153 (2024). 38461323 10.1038/s41467-024-45591-7 PMC10925021

[12] Schneeweiss, A. et al. Pertuzumab plus trastuzumab in combination with standard neoadjuvant anthracycline-containing and anthracycline-free chemotherapy regimens in patients with HER2-positive early breast cancer: a randomized phase II cardiac safety study (TRYPHAENA). *Ann. Oncol.* **24**, 2278–2284 (2013). 23704196 10.1093/annonc/mdt182

[13] van Ramshorst, M. S. et al. Neoadjuvant chemotherapy with or without anthracyclines in the presence of dual HER2 blockade for HER2-positive breast cancer (TRAIN-2): a multicentre, open-label, randomised, phase 3 trial. *Lancet Oncol.* **19**, 1630–1640 (2018). 30413379 10.1016/S1470-2045(18)30570-9

[14] Waks, A. G. et al. Dual HER2 inhibition: mechanisms of synergy, patient selection, and resistance. *Nat. Rev. Clin. Oncol.* **21**, 818–832 (2024). 39271787 10.1038/s41571-024-00939-2

[15] Ma, J. et al. Spatial molecular analyses reveal key features associated with response to KN026 in advanced HER2-positive breast cancer. *Br. J. Cancer* **134**, 685–696 (2025). 41366063 10.1038/s41416-025-03287-9 PMC12858834

[16] Chen, X.-C. et al. De-escalated neoadjuvant weekly nab-paclitaxel with trastuzumab and pertuzumab versus docetaxel, carboplatin, trastuzumab, and pertuzumab in patients with HER2-positive early breast cancer (HELEN-006): a multicentre, randomised, phase 3 trial. *Lancet Oncol.* **26**, 27–36 (2025). 39612919 10.1016/S1470-2045(24)00581-3

[17] Pérez-García, J. M. et al. Chemotherapy de-escalation using an 18F-FDG-PET-based pathological response-adapted strategy in patients with HER2-positive early breast cancer (PHERGain): a multicentre, randomised, open-label, non-comparative, phase 2 trial. *Lancet Oncol.* **22**, 858–871 (2021). 34019819 10.1016/S1470-2045(21)00122-4

[18] Gao, H.-F. et al. De-escalated neoadjuvant taxane plus trastuzumab and pertuzumab with or without carboplatin in HER2-positive early breast cancer (neoCARHP): a multicentre, open-label, randomised, phase 3 trial. *J. Clin. Oncol.* **43**, LBA500–LBA500 (2025).

[19] Li, J. J. et al. Efficacy and safety of neoadjuvant SHR-A1811 with or without pyrotinib in women with locally advanced or early HER2-positive breast cancer: a randomized, open-label, phase 2 trial. *Ann. Oncol.* **36**, 651–659 (2025). 10.1016/j.annonc.2025.02.011 40049447

[20] Wu, J. et al. Neoadjuvant pyrotinib, trastuzumab, and docetaxel for HER2-positive breast cancer (PHEDRA): a double-blind, randomized phase 3 trial. *BMC Med.* **20**, 498 (2022). 36575513 10.1186/s12916-022-02708-3 PMC9795751

[21] Lander, E. M. et al. Using the HER2/CEP17 FISH ratio to predict pathologic complete response following neoadjuvant anti-HER2 doublet therapy in HER2+ breast cancer. *Oncologist* **28**, 123–130 (2023). 36495309 10.1093/oncolo/oyac247 PMC9907044

[22] Bae, S. J. et al. Predictive markers of treatment response to neoadjuvant systemic therapy with dual HER2-blockade. *Cancers* **16**, 842 (2024). 38398233 10.3390/cancers16040842 PMC10886516

[23] Nuciforo, P. et al. A predictive model of pathologic response based on tumor cellularity and tumor-infiltrating lymphocytes (CelTIL) in HER2-positive breast cancer treated with chemo-free dual HER2 blockade. *Ann. Oncol.* **29**, 170–177 (2018). 29045543 10.1093/annonc/mdx647

[24] Llombart-Cussac, A. et al. HER2DX genomic assay in HER2-positive early breast cancer treated with trastuzumab and pertuzumab: a correlative analysis from the PHERGain phase II trial. *Clin. Cancer Res.* **30**, 4123–4130 (2024). 38995291 10.1158/1078-0432.CCR-24-0464 PMC11393543

[25] Guarneri, V. et al. HER2DX genomic test in HER2-positive/hormone receptor-positive breast cancer treated with neoadjuvant trastuzumab and pertuzumab: A correlative analysis from the PerELISA trial. *EBioMedicine* **85**, 104320 (2022). 36374768 10.1016/j.ebiom.2022.104320 PMC9626543

[26] Bueno-Muiño, C. et al. Assessment of a genomic assay in patients with ERBB2-positive breast cancer following neoadjuvant trastuzumab-based chemotherapy with or without pertuzumab. *JAMA Oncol.* **9**, 841–846 (2023). 37103916 10.1001/jamaoncol.2023.0187 PMC10141274

[27] Di Cosimo, S. et al. The 41-gene classifier TRAR predicts response of HER2 positive breast cancer patients in the NeoALTTO study. *Eur. J. Cancer* **118**, 1–9 (2019). 31284184 10.1016/j.ejca.2019.06.001

[28] Di Cosimo, S. et al. Plasma miRNA levels for predicting therapeutic response to neoadjuvant treatment in HER2-positive breast cancer: results from the NeoALTTO trial. *Clin. Cancer Res.* **25**, 3887–3895 (2019). 30814109 10.1158/1078-0432.CCR-18-2507

[29] Chen, J., Larsson, L., Swarbrick, A. & Lundeberg, J. Spatial landscapes of cancers: insights and opportunities. *Nat. Rev. Clin. Oncol.* **21**, 660–674 (2024). 39043872 10.1038/s41571-024-00926-7

[30] Goutsouliak, K. et al. Towards personalized treatment for early stage HER2-positive breast cancer. *Nat. Rev. Clin. Oncol.* **17**, 233–250 (2020). 31836877 10.1038/s41571-019-0299-9 PMC8023395

[31] Zhao, S. et al. Single-cell morphological and topological atlas reveals the ecosystem diversity of human breast cancer. *Nat. Commun.* **14**, 6796 (2023). 37880211 10.1038/s41467-023-42504-y PMC10600153

[32] Shamai, G. et al. Deep learning on histopathological images to predict breast cancer recurrence risk and chemotherapy benefit: a multicentre, model development and validation study. *Lancet Oncol.* **27**, 512–526 (2026). 41831466 10.1016/S1470-2045(25)00727-2 PMC12989675

[33] Ma, D. et al. Spatial determinants of antibody-drug conjugate SHR-A1811 efficacy in neoadjuvant treatment for HER2-positive breast cancer. *Cancer Cell* **43**, 1061–1075.e7 (2025). 40215979 10.1016/j.ccell.2025.03.017

[34] Swain, S. M. et al. Pertuzumab, trastuzumab, and docetaxel in HER2-positive metastatic breast cancer. *N. Engl. J. Med.* **372**, 724–734 (2015). 25693012 10.1056/NEJMoa1413513 PMC5584549

[35] Gianni, L. et al. 5-year analysis of neoadjuvant pertuzumab and trastuzumab in patients with locally advanced, inflammatory, or early-stage HER2-positive breast cancer (NeoSphere): a multicentre, open-label, phase 2 randomised trial. *Lancet Oncol.* **17**, 791–800 (2016). 27179402 10.1016/S1470-2045(16)00163-7

[36] Villacampa, G. et al. Association of HER2DX with pathological complete response and survival outcomes in HER2-positive breast cancer. *Ann. Oncol.* **34**, 783–795 (2023). 37302750 10.1016/j.annonc.2023.05.012 PMC10735273

[37] Waks, A. G. et al. Assessment of the HER2DX assay in patients with ERBB2-positive breast cancer treated with neoadjuvant paclitaxel, trastuzumab, and pertuzumab. *JAMA Oncol.* **9**, 835–840 (2023). 37103927 10.1001/jamaoncol.2023.0181 PMC10141272

[38] Prat, A. et al. A multivariable prognostic score to guide systemic therapy in early-stage HER2-positive breast cancer: a retrospective study with an external evaluation. *Lancet Oncol.* **21**, 1455–1464 (2020). 33152285 10.1016/S1470-2045(20)30450-2 PMC8140650

[39] Mao, N. et al. A multimodal and fully automated system for prediction of pathological complete response to neoadjuvant chemotherapy in breast cancer. *Sci. Adv.* **11**, eadr1576 (2025). 40305609 10.1126/sciadv.adr1576 PMC12042891

[40] Urso, L. et al. 18F-FDG PET/CT radiomic analysis and artificial intelligence to predict pathological complete response after neoadjuvant chemotherapy in breast cancer patients. *Radio. Med.* **130**, 543–554 (2025). 10.1007/s11547-025-01958-4 PMC12008070 39875749

[41] Nitz, U. et al. De-escalated neoadjuvant pertuzumab plus trastuzumab therapy with or without weekly paclitaxel in HER2-positive, hormone receptor-negative, early breast cancer (WSG-ADAPT-HER2+/HR-): survival outcomes from a multicentre, open-label, randomised, phase 2 trial. *Lancet Oncol.* **23**, 625–635 (2022). 35405088 10.1016/S1470-2045(22)00159-0

[42] Abdel-Razeq, H. De-escalating treatment strategies for patients with human epidermal growth factor receptor-2 (HER2)-positive early-stage breast cancer. *Cancers* **16**, 3478 (2024). 39456572 10.3390/cancers16203478 PMC11506701

[43] Choong, G. M., Cullen, G. D. & O’Sullivan, C. C. Evolving standards of care and new challenges in the management of HER2-positive breast cancer. *CA Cancer J. Clin.* **70**, 355–374 (2020). 32813307 10.3322/caac.21634

[44] Schettini, F. & Prat, A. Dissecting the biological heterogeneity of HER2-positive breast cancer. *Breast* **59**, 339–350 (2021). 34392185 10.1016/j.breast.2021.07.019 PMC8374722

[45] Li, Z. et al. HER2 heterogeneity and treatment response-associated profiles in HER2-positive breast cancer in the NCT02326974 clinical trial. *J. Clin. Invest.* **134**, e176454 (2024). 38300710 10.1172/JCI176454 PMC10977978

[46] Mosele, F. et al. Trastuzumab deruxtecan in metastatic breast cancer with variable HER2 expression: the phase 2 DAISY trial. *Nat. Med.* **29**, 2110–2120 (2023). 37488289 10.1038/s41591-023-02478-2 PMC10427426

[47] Valenza, C. et al. Targeting HER2 heterogeneity in breast and gastrointestinal cancers. *Trends Cancer* **10**, 113–123 (2024). 38008666 10.1016/j.trecan.2023.11.001

[48] Ogitani, Y., Hagihara, K., Oitate, M., Naito, H. & Agatsuma, T. Bystander killing effect of DS-8201a, a novel anti-human epidermal growth factor receptor 2 antibody-drug conjugate, in tumors with human epidermal growth factor receptor 2 heterogeneity. *Cancer Sci.* **107**, 1039–1046 (2016). 27166974 10.1111/cas.12966 PMC4946713

[49] Tsao, L.-C. et al. Effective extracellular payload release and immunomodulatory interactions govern the therapeutic effect of trastuzumab deruxtecan (T-DXd). *Nat. Commun.* **16**, 3167 (2025). 40175391 10.1038/s41467-025-58266-8 PMC11965298

[50] Ma, F. et al. Pyrotinib versus placebo in combination with trastuzumab and docetaxel as first line treatment in patients with HER2 positive metastatic breast cancer (PHILA): randomised, double blind, multicentre, phase 3 trial. *BMJ* **383**, e076065 (2023). 37907210 10.1136/bmj-2023-076065 PMC10616786

[51] Wang, X. et al. Spatial interplay patterns of cancer nuclei and tumor-infiltrating lymphocytes (TILs) predict clinical benefit for immune checkpoint inhibitors. *Sci. Adv.* **8**, eabn3966 (2022). 35648850 10.1126/sciadv.abn3966 PMC9159577

[52] McNamara, K. L. et al. Spatial proteomic characterization of HER2-positive breast tumors through neoadjuvant therapy predicts response. *Nat. Cancer* **2**, 400–413 (2021). 34966897 10.1038/s43018-021-00190-z PMC8713949

[53] Vono, M. et al. Neutrophils acquire the capacity for antigen presentation to memory CD4+ T cells in vitro and ex vivo. *Blood* **129**, 1991–2001 (2017). 28143882 10.1182/blood-2016-10-744441 PMC5383872

[54] Fanger, N. A. et al. Activation of human T cells by major histocompatability complex class II expressing neutrophils: proliferation in the presence of superantigen, but not tetanus toxoid. *Blood* **89**, 4128–4135 (1997). 9166855

[55] Lad, M. et al. Glioblastoma induces the recruitment and differentiation of dendritic-like “hybrid” neutrophils from skull bone marrow. *Cancer Cell* **42**, 1549–1569.e16 (2024). 39255776 10.1016/j.ccell.2024.08.008 PMC11446475

[56] Wu, Y. et al. Neutrophil profiling illuminates anti-tumor antigen-presenting potency. *Cell* **187**, 1422–1439.e24 (2024). 38447573 10.1016/j.cell.2024.02.005

[57] Collins, G. S., Reitsma, J. B., Altman, D. G. & Moons, K. G. M. Transparent reporting of a multivariable prediction model for individual prognosis or diagnosis (TRIPOD): the TRIPOD statement. *BMC Med.* **13**, 1 (2015). 25563062 10.1186/s12916-014-0241-z PMC4284921

[58] Wolff, A. C. et al. Human epidermal growth factor receptor 2 testing in breast cancer: ASCO-College of American Pathologists Guideline Update. *J. Clin. Oncol.* **41**, 3867–3872 (2023). 37284804 10.1200/JCO.22.02864

[59] Graham, S. et al. Hover-Net: simultaneous segmentation and classification of nuclei in multi-tissue histology images. *Med. Image Anal.* **58**, 101563 (2019). 31561183 10.1016/j.media.2019.101563

[60] Gamper, J., Alemi Koohbanani, N., Benet, K., Khuram, A. & Rajpoot, N. *PanNuke: An Open Pan-Cancer Histology Dataset for Nuclei Instance Segmentation and Classification* (Springer International Publishing, 2019).

[61] Verma, R. et al. MoNuSAC2020: a multi-organ nuclei segmentation and classification challenge. *IEEE Trans. Med. Imaging* **40**, 3413–3423 (2021). 34086562 10.1109/TMI.2021.3085712

[62] Li, H. et al. Segment Membranes and Nuclei from Histopathological Images via Nuclei Point-Level Supervision. In *Medical Image Computing and Computer Assisted Intervention – MICCAI 2023* (eds. Greenspan, H., et al.) 539–548 (Springer Nature, 2023).

[63] Kratz, J. R. et al. A practical molecular assay to predict survival in resected non-squamous, non-small-cell lung cancer: development and international validation studies. *Lancet* **379**, 823–832 (2012). 22285053 10.1016/S0140-6736(11)61941-7 PMC3294002

[64] Collins, G. S. et al. TRIPOD+AI statement: updated guidance for reporting clinical prediction models that use regression or machine learning methods. *BMJ* **385**, e078378 (2024). 38626948 10.1136/bmj-2023-078378 PMC11019967

[65] Lundberg, S. M. & Lee, S.-I. A unified approach to interpreting model predictions. In *Proc. 31st International Conference on Neural Information Processing Systems* 4768–4777 (Curran Associates Inc., 2017)

