# Supplementary material for: Spatially interpretable artificial intelligence framework to tailored neoadjuvant dual HER2 blockade in HER2-positive breast cancer
Source: Signal Transduct Target Ther. 2026 Jun 19;11:241. doi: 10.1038/s41392-026-02734-0 (PMC13280384; doi:10.1038/s41392-026-02734-0)
Supplement: Supplementary file 1 — Supplementary Materials [file 41392_2026_2734_MOESM1_ESM.docx]

Supplementary Materials for

Spatially interpretable artificial intelligence framework to tailored neoadjuvant dual HER2 blockade in HER2-positive breast cancer

Xiang-Rong Wu, Hong Lv, Shen Zhao, Xiao-Hua Zeng, Lei-Jie Dai, Yu-Zheng Xu, Yu-Wei Li, Zi-Yu Qiu, Ji-Ting Huang, Ning-Ning Zhang, Li Chen, Min He, Yi-Zhi Zhao, Lin Yang,
Tong Zhou, Jun-Jie Li, Jiong Wu, Yi-Zhou Jiang, Wen-Tao Yang, Gen-Hong Di,
Zhi-Ming Shao, Ding Ma

Correspondence to: dma09@fudan.edu.cn

**This PDF file includes:**

Supplementary Figs. 1-5

Supplementary Table 1

**
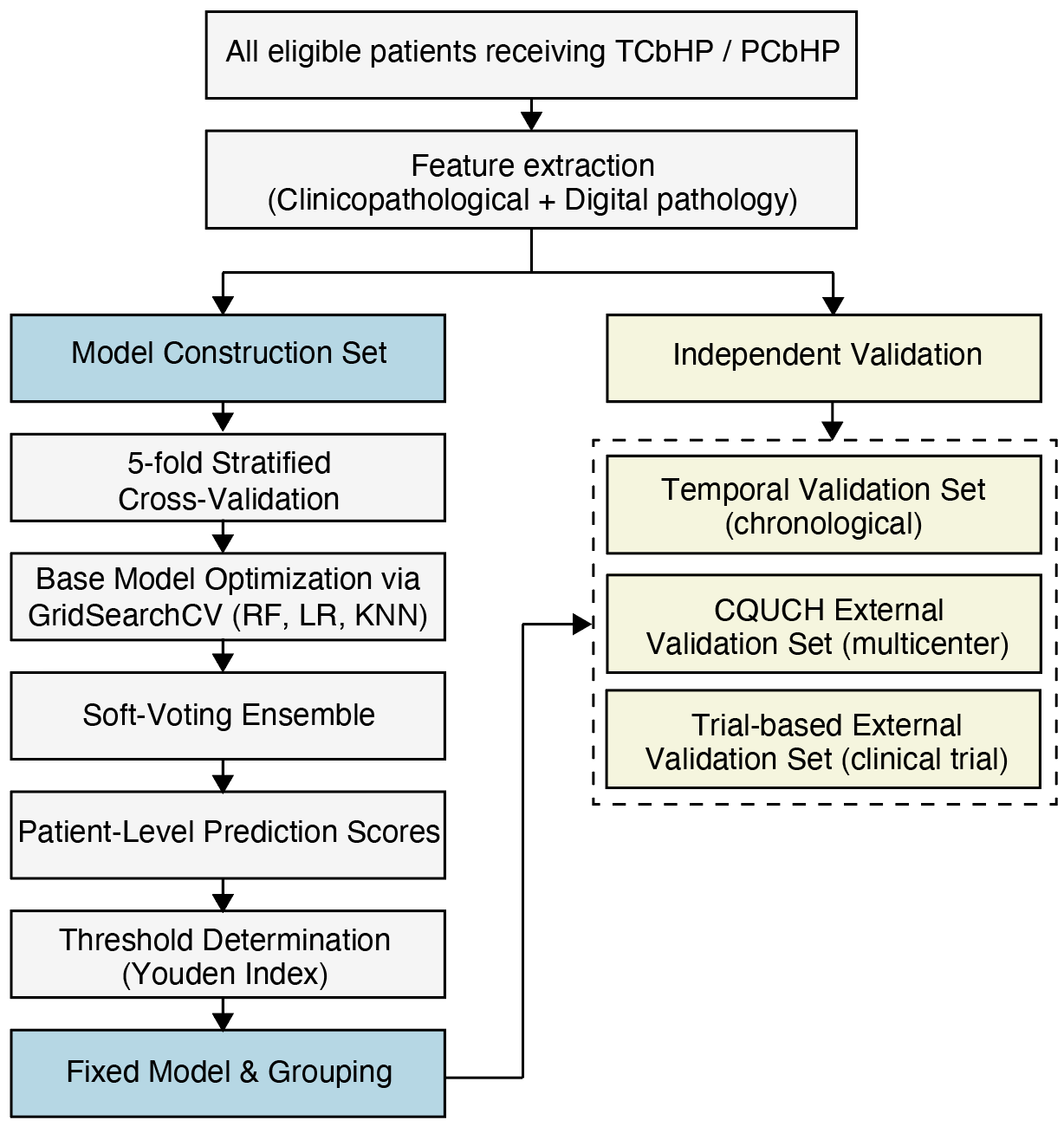
**

**Supplementary Fig. 1. Data splitting, model construction, and validation workflow for the HER2-LADDER framework.**


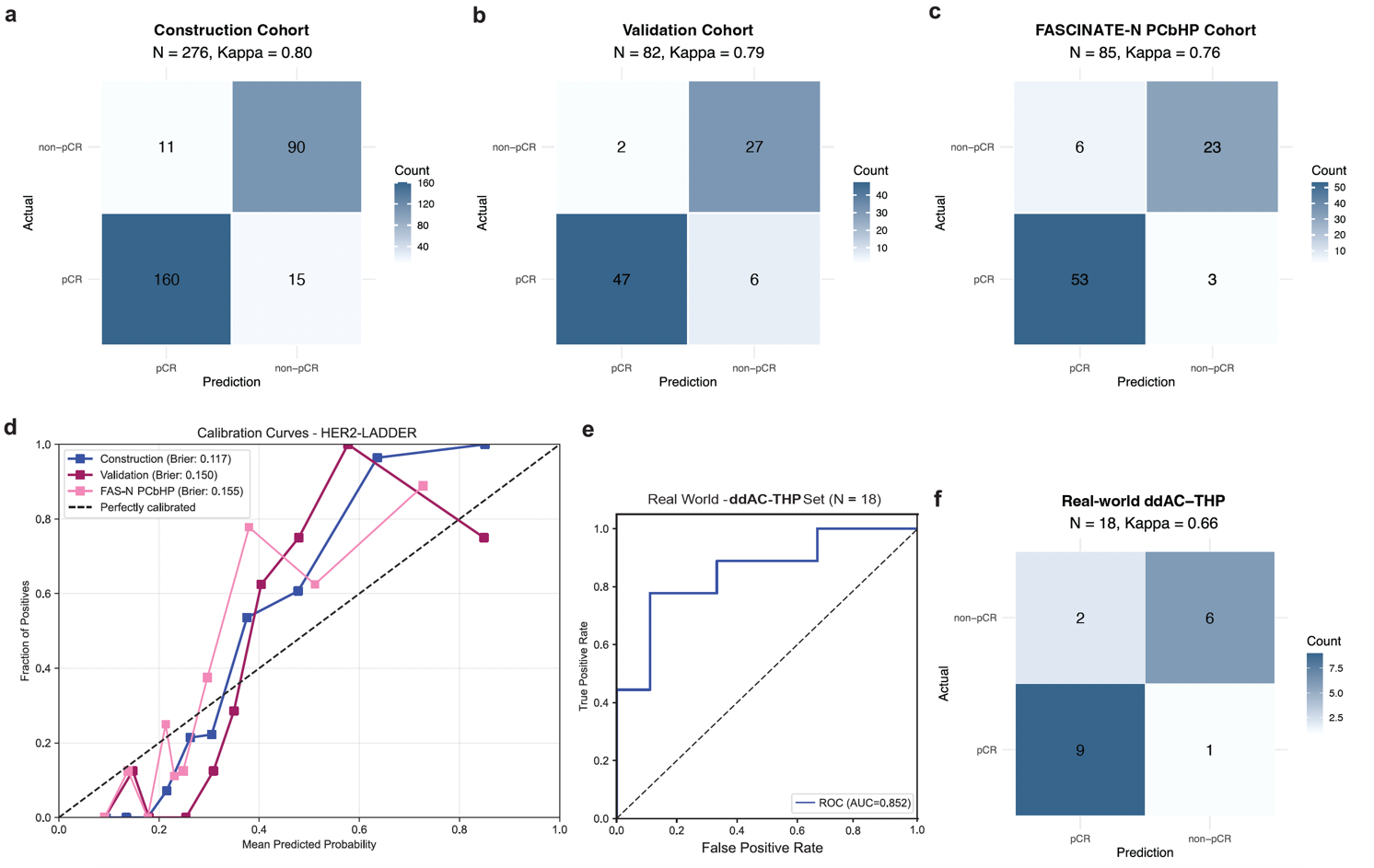


Supplementary Fig. 2. Model performance evaluation across multiple cohorts.

**a–c,** Confusion matrix analyses illustrating the agreement between predicted and observed pathological complete response (pCR) outcomes across the model construction cohort (a), temporal validation cohort (b), and the FASCINATE-N PCbHP clinical trial cohort (c). Consistent classification accuracy and reproducibility were observed, with Cohen’s kappa coefficients of 0.80, 0.79, and 0.76, respectively.

**d,** Calibration curves showing strong concordance between predicted and observed probabilities across all cohorts. The corresponding Brier scores were 0.117 for the model construction set, 0.150 for the temporal validation set, and 0.155 for the FASCINATE-N PCbHP cohort, indicating reliable probabilistic calibration.

**e–f,** External validation in a real-world neoadjuvant ddAC-THP cohort (n = 18) demonstrated stable discriminative performance (AUC = 0.852) and classification agreement (Cohen’s kappa = 0.66). Collectively, these findings confirm the robustness, calibration reliability, and generalizability of the HER2-LADDER model across diverse clinical contexts.

**
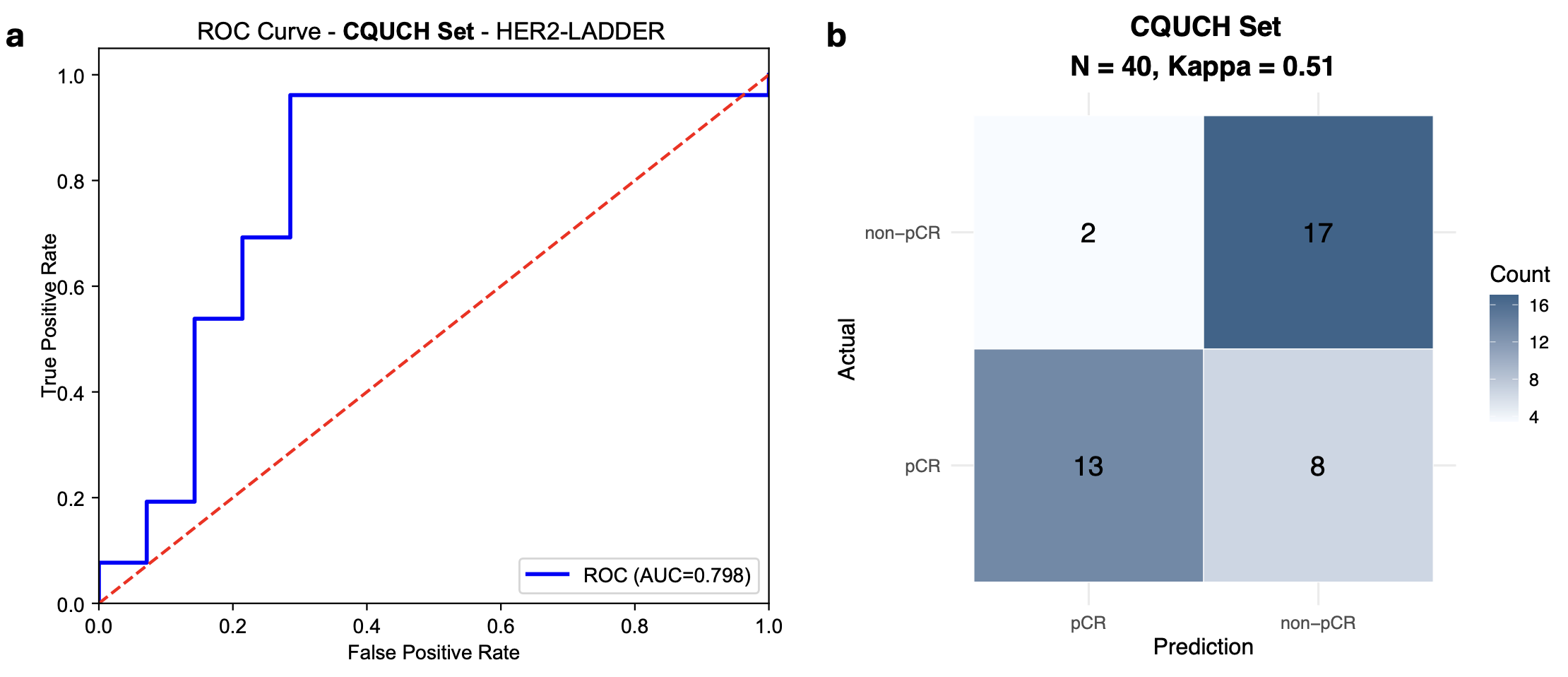
**

Supplementary Fig. 3. Independent external validation of HER2-LADDER in the Chongqing University Cancer Hospital (CQUCH) cohort.

**a,** ROC curve of HER2-LADDER applied to an external cohort from CQUCH.

**b,** Confusion matrix of predicted versus observed treatment.


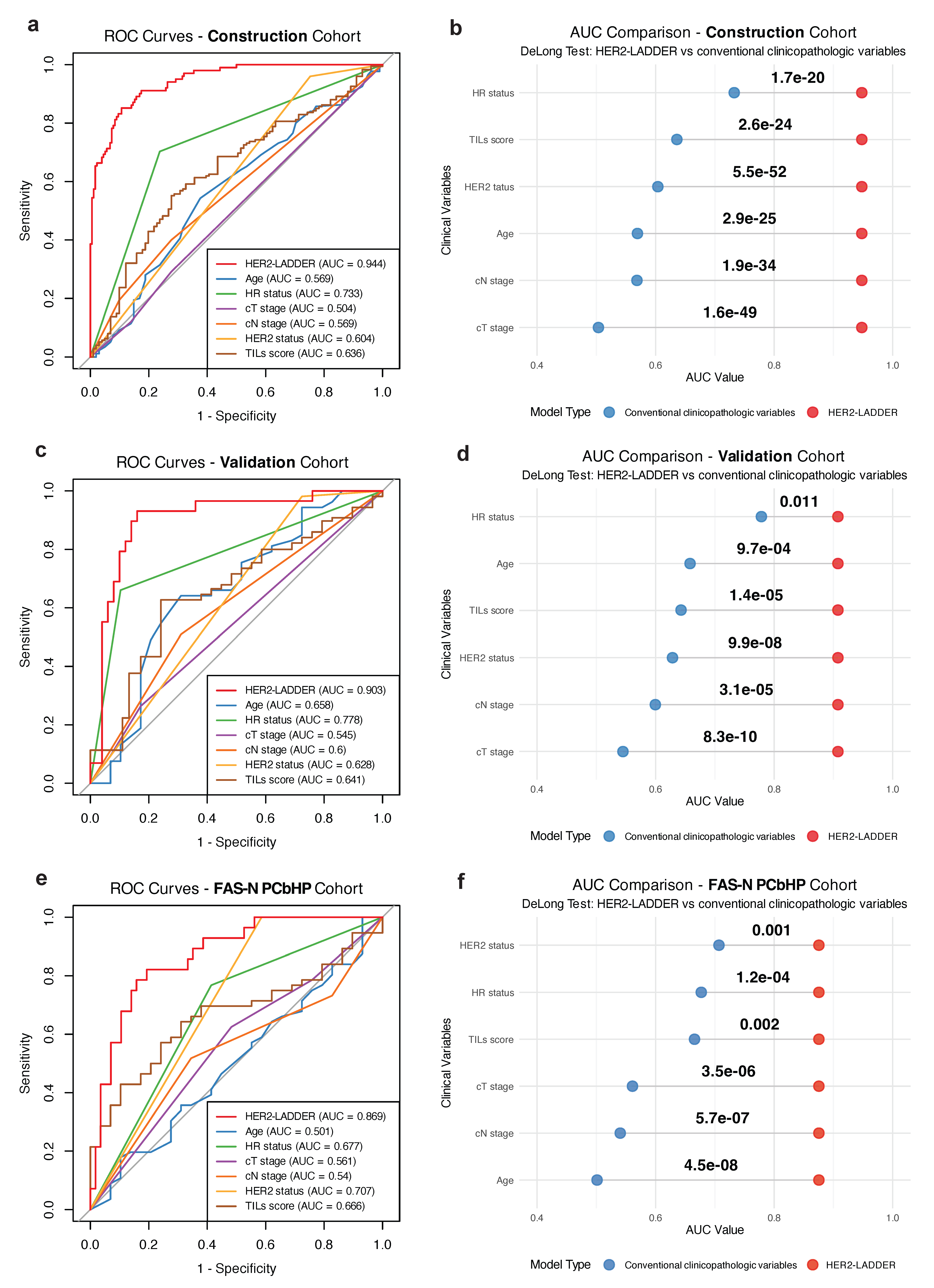


Supplementary Fig. 4. Comparative predictive performance of HER2-LADDER and conventional clinicopathologic variables.

**a,c,e,** ROC curves comparing HER2-LADDER with individual clinicopathologic predictors in the model construction cohort, validation cohort, and trial-based external cohort, respectively.

**b,d,f,** AUC comparisons between HER2-LADDER and conventional clinicopathologic variables, with statistical significance assessed using DeLong tests. HER2-LADDER consistently demonstrated significantly higher AUCs across all cohorts, indicating superior discrimination ability.


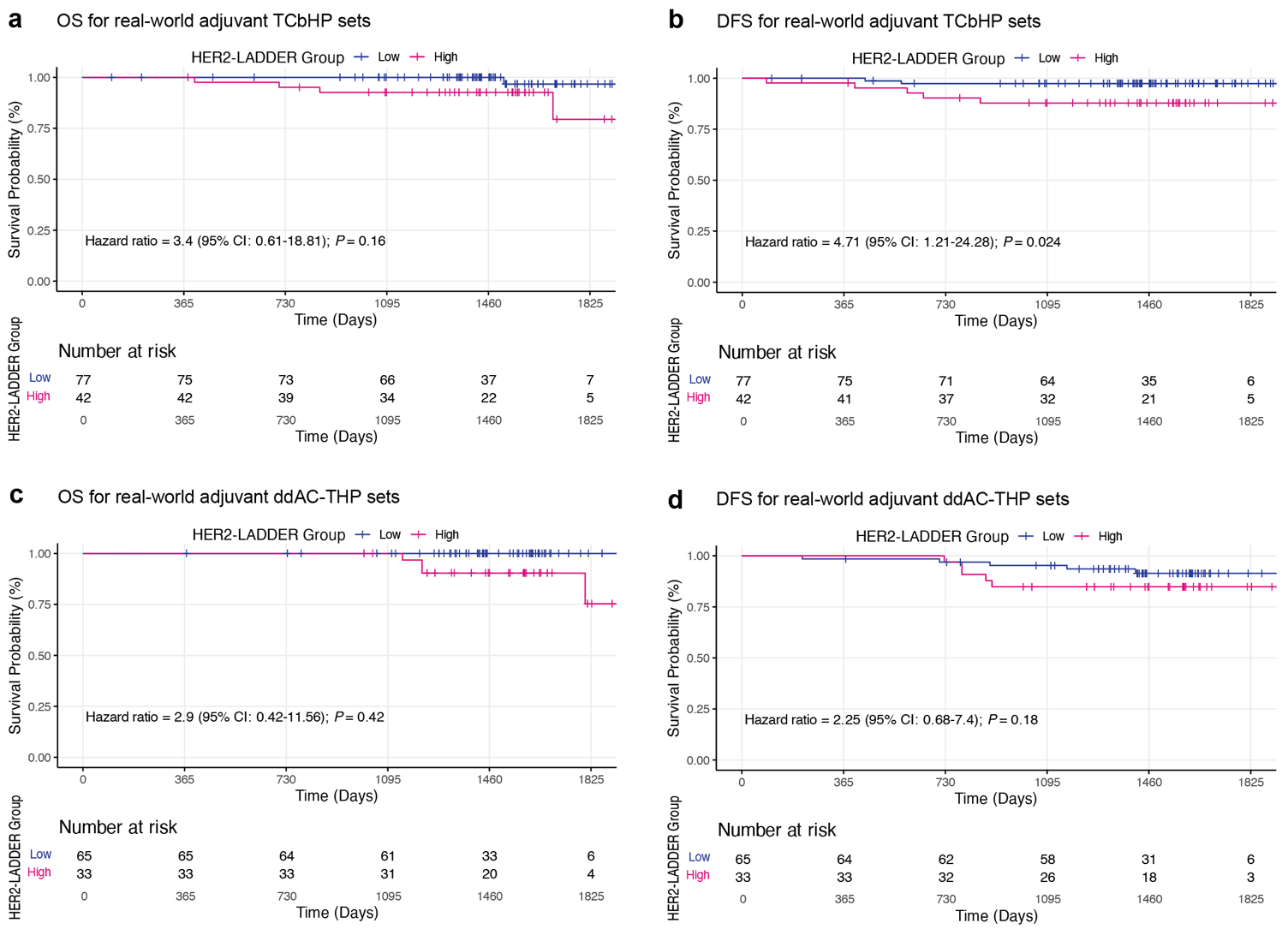


Supplementary Fig. 5. Overall survival and disease-free survival analysis stratified by HER2-LADDER score in the adjuvant TCbHP and ddAC-THP subgroups.

**a,** overall survival in patients receiving adjuvant TCbHP.

**b,** disease-free survival in patients receiving adjuvant TCbHP.

**c,** overall survival in patients receiving adjuvant ddAC-THP.

**d,** disease-free survival in patients receiving adjuvant ddAC-THP.

Supplementary Table 1. Summary of spatially resolved single-cell features extracted using the sc-MTOP framework from H&E and HER2 IHC whole slide images.

| **Variable name** | **Variable type** | **Explanation** | **Identification basis** |
| --- | --- | --- | --- |
| Pi_Tumor_in_all_cells | Cell proportion | Proportion of tumor cells among all cells | H&E staining slide |
| Pi_Lymph_in_all_cells | Cell proportion | Proportion of lymphocytes among all cells | H&E staining slide |
| Pi_Macro_in_all_cells | Cell proportion | Proportion of macrophages among all cells | H&E staining slide |
| Pi_Neutro_in_all_cells | Cell proportion | Proportion of neutrophils among all cells | H&E staining slide |
| Pi_Stroma_in_all_cells | Cell proportion | Proportion of stroma among all cells | H&E staining slide |
| Pi_Lymph_in_TME_cells | Cell proportion | Proportion of lymphocytes within tumor microenvironment cells | H&E staining slide |
| Pi_Macro_in_TME_cells | Cell proportion | Proportion of macrophages within tumor microenvironment cells | H&E staining slide |
| Pi_Neutro_in_TME_cells | Cell proportion | Proportion of neutrophils within tumor microenvironment cells | H&E staining slide |
| Pi_Stroma_in_TME_cells | Cell proportion | Proportion of stroma within tumor microenvironment cells | H&E staining slide |
| Tumor_Tumor_minEdgeLength | Spatial distance | Minimum edge length between tumor and tumor cells | H&E staining slide |
| Tumor_Tumor_meanEdgeLength | Spatial distance | Mean edge length between tumor and tumor cells | H&E staining slide |
| Tumor_Tumor_Nsubgraph | Cluster topology | Number of subgraphs (clusters) formed between tumor and tumor cells | H&E staining slide |
| Tumor_Tumor_Degrees | Cellular connectivity | Average degree of tumor and tumor cells in graph network | H&E staining slide |
| Tumor_Neutro_minEdgeLength | Spatial distance | Minimum edge length between tumor cells and neutrophils | H&E staining slide |
| Tumor_Neutro_meanEdgeLength | Spatial distance | Mean edge length between tumor cells and neutrophils | H&E staining slide |
| Tumor_Neutro_Nsubgraph | Cluster topology | Number of subgraphs (clusters) formed between tumor and neutrophils | H&E staining slide |
| Tumor_Neutro_Degrees | Cellular connectivity | Average degree of tumor cells and neutrophils in graph network | H&E staining slide |
| Tumor_Lymph_minEdgeLength | Spatial distance | Minimum edge length between tumor cells and lymphocytes | H&E staining slide |
| Tumor_Lymph_meanEdgeLength | Spatial distance | Mean edge length between tumor cells and lymphocytes | H&E staining slide |
| Tumor_Lymph_Nsubgraph | Cluster topology | Number of subgraphs (clusters) formed between tumor cells and lymphocytes | H&E staining slide |
| Tumor_Lymph_Degrees | Cellular connectivity | Average degree of tumor cells and lymphocytes in graph network | H&E staining slide |
| Tumor_Macro_minEdgeLength | Spatial distance | Minimum edge length between tumor cells and macrophages | H&E staining slide |
| Tumor_Macro_meanEdgeLength | Spatial distance | Mean edge length between tumor cells and macrophages | H&E staining slide |
| Tumor_Macro_Nsubgraph | Cluster topology | Number of subgraphs (clusters) formed between tumor cells and macrophages | H&E staining slide |
| Tumor_Macro_Degrees | Cellular connectivity | Average degree of tumor cells and macrophages in graph network | H&E staining slide |
| Tumor_Stroma_minEdgeLength | Spatial distance | Minimum edge length between tumor cells and stroma cells | H&E staining slide |
| Tumor_Stroma_meanEdgeLength | Spatial distance | Mean edge length between tumor cells and stroma cells | H&E staining slide |
| Tumor_Stroma_Nsubgraph | Cluster topology | Number of subgraphs (clusters) formed between tumor cells and stroma cells | H&E staining slide |
| Tumor_Stroma_Degrees | Cellular connectivity | Average degree of tumor cells and stroma cells in graph network | H&E staining slide |
| Neutro_Neutro_minEdgeLength | Spatial distance | Minimum edge length between neutrophils and neutrophils | H&E staining slide |
| Neutro_Neutro_meanEdgeLength | Spatial distance | Mean edge length between neutrophils and neutrophils | H&E staining slide |
| Neutro_Neutro_Nsubgraph | Cluster topology | Number of subgraphs (clusters) formed between neutrophils and neutrophils | H&E staining slide |
| Neutro_Neutro_Degrees | Cellular connectivity | Average degree of neutrophils and neutrophils in graph network | H&E staining slide |
| Neutro_Lymph_minEdgeLength | Spatial distance | Minimum edge length between neutrophils and lymphocytes | H&E staining slide |
| Neutro_Lymph_meanEdgeLength | Spatial distance | Mean edge length between neutrophils and lymphocytes | H&E staining slide |
| Neutro_Lymph_Nsubgraph | Cluster topology | Number of subgraphs (clusters) formed between neutrophils and lymphocytes | H&E staining slide |
| Neutro_Lymph_Degrees | Cellular connectivity | Average degree of neutrophils and lymphocytes in graph network | H&E staining slide |
| Neutro_Macro_minEdgeLength | Spatial distance | Minimum edge length between neutrophils and macrophages | H&E staining slide |
| Neutro_Macro_meanEdgeLength | Spatial distance | Mean edge length between neutrophils and macrophages | H&E staining slide |
| Neutro_Macro_Nsubgraph | Cluster topology | Number of subgraphs (clusters) formed between neutrophils and macrophages | H&E staining slide |
| Neutro_Macro_Degrees | Cellular connectivity | Average degree of neutrophils and macrophages in graph network | H&E staining slide |
| Neutro_Stroma_minEdgeLength | Spatial distance | Minimum edge length between neutrophils and stroma cells | H&E staining slide |
| Neutro_Stroma_meanEdgeLength | Spatial distance | Mean edge length between neutrophils and stroma cells | H&E staining slide |
| Neutro_Stroma_Nsubgraph | Cluster topology | Number of subgraphs (clusters) formed between neutrophils and stroma cells | H&E staining slide |
| Neutro_Stroma_Degrees | Cellular connectivity | Average degree of neutrophils and stroma cells in graph network | H&E staining slide |
| Lymph_Lymph_minEdgeLength | Spatial distance | Minimum edge length between lymphocytes and lymphocytes | H&E staining slide |
| Lymph_Lymph_meanEdgeLength | Spatial distance | Mean edge length between lymphocytes and lymphocytes | H&E staining slide |
| Lymph_Lymph_Nsubgraph | Cluster topology | Number of subgraphs (clusters) formed between lymphocytes and lymphocytes | H&E staining slide |
| Lymph_Lymph_Degrees | Cellular connectivity | Average degree of lymphocytes and lymphocytes in graph network | H&E staining slide |
| Lymph_Macro_minEdgeLength | Spatial distance | Minimum edge length between lymphocytes and macrophages | H&E staining slide |
| Lymph_Macro_meanEdgeLength | Spatial distance | Mean edge length between lymphocytes and macrophages | H&E staining slide |
| Lymph_Macro_Nsubgraph | Cluster topology | Number of subgraphs (clusters) formed between lymphocytes and macrophages | H&E staining slide |
| Lymph_Macro_Degrees | Cellular connectivity | Average degree of lymphocytes and macrophages in graph network | H&E staining slide |
| Lymph_Stroma_minEdgeLength | Spatial distance | Minimum edge length between lymphocytes and stroma cells | H&E staining slide |
| Lymph_Stroma_meanEdgeLength | Spatial distance | Mean edge length between lymphocytes and stroma cells | H&E staining slide |
| Lymph_Stroma_Nsubgraph | Cluster topology | Number of subgraphs (clusters) formed between lymphocytes and stroma cells | H&E staining slide |
| Lymph_Stroma_Degrees | Cellular connectivity | Average degree of lymphocytes and stroma cells in graph network | H&E staining slide |
| Macro_Macro_minEdgeLength | Spatial distance | Minimum edge length between macrophages and macrophages | H&E staining slide |
| Macro_Macro_meanEdgeLength | Spatial distance | Mean edge length between macrophages and macrophages | H&E staining slide |
| Macro_Macro_Nsubgraph | Cluster topology | Number of subgraphs (clusters) formed between macrophages and macrophages | H&E staining slide |
| Macro_Macro_Degrees | Cellular connectivity | Average degree of macrophages and macrophages in graph network | H&E staining slide |
| Macro_Stroma_minEdgeLength | Spatial distance | Minimum edge length between macrophages and stroma cells | H&E staining slide |
| Macro_Stroma_meanEdgeLength | Spatial distance | Mean edge length between macrophages and stroma cells | H&E staining slide |
| Macro_Stroma_Nsubgraph | Cluster topology | Number of subgraphs (clusters) formed between macrophages and stroma cells | H&E staining slide |
| Macro_Stroma_Degrees | Cellular connectivity | Average degree of macrophages and stroma cells in graph network | H&E staining slide |
| Stroma_Stroma_minEdgeLength | Spatial distance | Minimum edge length between stroma and stroma cells | H&E staining slide |
| Stroma_Stroma_meanEdgeLength | Spatial distance | Mean edge length between stroma and stroma cells | H&E staining slide |
| Stroma_Stroma_Nsubgraph | Cluster topology | Number of subgraphs (clusters) formed between stroma and stroma cells | H&E staining slide |
| Stroma_Stroma_Degrees | Cellular connectivity | Average degree of stroma and stroma cells in graph network | H&E staining slide |
| Pi_HER2strong_complete_in_HER2strong | Cell proportion | Proportion of HER2-strong-posittive_complete among HER2 strong-positive cells | HER2 IHC staining slide |
| Pi_HER2strong_incomplete_in_HER2strong | Cell proportion | Proportion of HER2-strong-posittive_incomplete among HER2 strong-positive cells | HER2 IHC staining slide |
| Pi_HER2strong_complete_in_tumor_cells | Cell proportion | Proportion of HER2-strong-posittive_complete among tumor cells | HER2 IHC staining slide |
| Pi_HER2strong_incomplete_in_tumor_cells | Cell proportion | Proportion of HER2-strong-posittive_incomplete among tumor cells | HER2 IHC staining slide |
| Pi_HER2strong_in_tumor_cells | Cell proportion | Proportion of HER2-strong-posittive among tumor cells | HER2 IHC staining slide |
| Pi_HER2weak_complete_in_HER2weak | Cell proportion | Proportion of HER2-weak-posittive_complete among HER2 weak-positive cells | HER2 IHC staining slide |
| Pi_HER2weak_incomplete_in_HER2weak | Cell proportion | Proportion of HER2-weak-posittive_incomplete among HER2 weak-positive cells | HER2 IHC staining slide |
| Pi_HER2weak_complete_in_tumor_cells | Cell proportion | Proportion of HER2-weak-posittive_complete among tumor cells | HER2 IHC staining slide |
| Pi_HER2weak_incomplete_in_tumor_cells | Cell proportion | Proportion of HER2-weak-posittive_incomplete among tumor cells | HER2 IHC staining slide |
| Pi_HER2weak_in_tumor_cells | Cell proportion | Proportion of HER2-weak-posittive among tumor cells | HER2 IHC staining slide |
| HER2strong_complete_HER2strong_complete_minEdgeLength | Spatial distance | Minimum edge length between complete HER2-strong-positive and complete HER2-strong-positive cells | HER2 IHC staining slide |
| HER2strong_complete_HER2strong_complete_meanEdgeLength | Spatial distance | Mean edge length between complete HER2-strong-positive and complete HER2-strong-positive cells | HER2 IHC staining slide |
| HER2strong_complete_HER2strong_complete_Nsubgraph | Cluster topology | Number of subgraphs (clusters) formed between complete HER2-strong-positive and complete HER2-strong-positive cells | HER2 IHC staining slide |
| HER2strong_complete_HER2strong_complete_Degrees | Cellular connectivity | Average degree of complete HER2-strong-positive and complete HER2-strong-positive cells in graph network | HER2 IHC staining slide |
| HER2strong_complete_HER2strong_incomplete_minEdgeLength | Spatial distance | Minimum edge length between complete HER2-strong-positive and incomplete HER2-strong-positive cells | HER2 IHC staining slide |
| HER2strong_complete_HER2strong_incomplete_meanEdgeLength | Spatial distance | Mean edge length between complete HER2-strong-positive and incomplete HER2-strong-positive cells | HER2 IHC staining slide |
| HER2strong_complete_HER2strong_incomplete_Nsubgraph | Cluster topology | Number of subgraphs (clusters) formed between complete HER2-strong-positive and incomplete HER2-strong-positive cells | HER2 IHC staining slide |
| HER2strong_complete_HER2strong_incomplete_Degrees | Cellular connectivity | Average degree of complete HER2-strong-positive and incomplete HER2-strong-positive cells in graph network | HER2 IHC staining slide |
| HER2strong_complete_HER2weak_complete_minEdgeLength | Spatial distance | Minimum edge length between complete HER2-strong-positive and complete HER2-weak-positive cells | HER2 IHC staining slide |
| HER2strong_complete_HER2weak_complete_meanEdgeLength | Spatial distance | Mean edge length between complete HER2-strong-positive and complete HER2-weak-positive cells | HER2 IHC staining slide |
| HER2strong_complete_HER2weak_complete_Nsubgraph | Cluster topology | Number of subgraphs (clusters) formed between complete HER2-strong-positive and complete HER2-weak-positive cells | HER2 IHC staining slide |
| HER2strong_complete_HER2weak_complete_Degrees | Cellular connectivity | Average degree of complete HER2-strong-positive and complete HER2-weak-positive cells in graph network | HER2 IHC staining slide |
| HER2strong_complete_HER2weak_incomplete_minEdgeLength | Spatial distance | Minimum edge length between complete HER2-strong-positive and incomplete HER2-weak-positive cells | HER2 IHC staining slide |
| HER2strong_complete_HER2weak_incomplete_meanEdgeLength | Spatial distance | Mean edge length between complete HER2-strong-positive and incomplete HER2-weak-positive cells | HER2 IHC staining slide |
| HER2strong_complete_HER2weak_incomplete_Nsubgraph | Cluster topology | Number of subgraphs (clusters) formed between complete HER2-strong-positive and incomplete HER2-weak-positive cells | HER2 IHC staining slide |
| HER2strong_complete_HER2weak_incomplete_Degrees | Cellular connectivity | Average degree of complete HER2-strong-positive and incomplete HER2-weak-positive cells in graph network | HER2 IHC staining slide |
| HER2strong_complete_HER2null_minEdgeLength | Spatial distance | Minimum edge length between complete HER2-strong-positive and HER2-null cells | HER2 IHC staining slide |
| HER2strong_complete_HER2null_meanEdgeLength | Spatial distance | Mean edge length between complete HER2-strong-positive and HER2-null cells | HER2 IHC staining slide |
| HER2strong_complete_HER2null_Nsubgraph | Cluster topology | Number of subgraphs (clusters) formed between complete HER2-strong-positive and HER2-null cells | HER2 IHC staining slide |
| HER2strong_complete_HER2null_Degrees | Cellular connectivity | Average degree of complete HER2-strong-positive and HER2-null cells in graph network | HER2 IHC staining slide |
| HER2strong_incomplete_HER2strong_incomplete_minEdgeLength | Spatial distance | Minimum edge length between incomplete HER2-strong-positive and incomplete HER2-strong-positive cells | HER2 IHC staining slide |
| HER2strong_incomplete_HER2strong_incomplete_meanEdgeLength | Spatial distance | Mean edge length between incomplete HER2-strong-positive and incomplete HER2-strong-positive cells | HER2 IHC staining slide |
| HER2strong_incomplete_HER2strong_incomplete_Nsubgraph | Cluster topology | Number of subgraphs (clusters) formed between incomplete HER2-strong-positive and incomplete HER2-strong-positive cells | HER2 IHC staining slide |
| HER2strong_incomplete_HER2strong_incomplete_Degrees | Cellular connectivity | Average degree of incomplete HER2-strong-positive and incomplete HER2-strong-positive cells in graph network | HER2 IHC staining slide |
| HER2strong_incomplete_HER2weak_complete_minEdgeLength | Spatial distance | Minimum edge length between incomplete HER2-strong-positive and complete HER2-weak-positive cells | HER2 IHC staining slide |
| HER2strong_incomplete_HER2weak_complete_meanEdgeLength | Spatial distance | Mean edge length between incomplete HER2-strong-positive and complete HER2-weak-positive cells | HER2 IHC staining slide |
| HER2strong_incomplete_HER2weak_complete_Nsubgraph | Cluster topology | Number of subgraphs (clusters) formed between incomplete HER2-strong-positive and complete HER2-weak-positive cells | HER2 IHC staining slide |
| HER2strong_incomplete_HER2weak_complete_Degrees | Cellular connectivity | Average degree of incomplete HER2-strong-positive and complete HER2-weak-positive cells in graph network | HER2 IHC staining slide |
| HER2strong_incomplete_HER2weak_incomplete_minEdgeLength | Spatial distance | Minimum edge length between incomplete HER2-strong-positive and incomplete HER2-weak-positive cells | HER2 IHC staining slide |
| HER2strong_incomplete_HER2weak_incomplete_meanEdgeLength | Spatial distance | Mean edge length between incomplete HER2-strong-positive and incomplete HER2-weak-positive cells | HER2 IHC staining slide |
| HER2strong_incomplete_HER2weak_incomplete_Nsubgraph | Cluster topology | Number of subgraphs (clusters) formed between incomplete HER2-strong-positive and incomplete HER2-weak-positive cells | HER2 IHC staining slide |
| HER2strong_incomplete_HER2weak_incomplete_Degrees | Cellular connectivity | Average degree of incomplete HER2-strong-positive and incomplete HER2-weak-positive cells in graph network | HER2 IHC staining slide |
| HER2strong_incomplete_HER2null_minEdgeLength | Spatial distance | Minimum edge length between incomplete HER2-strong-positive and HER2-null cells | HER2 IHC staining slide |
| HER2strong_incomplete_HER2null_meanEdgeLength | Spatial distance | Mean edge length between incomplete HER2-strong-positive and HER2-null cells | HER2 IHC staining slide |
| HER2strong_incomplete_HER2null_Nsubgraph | Cluster topology | Number of subgraphs (clusters) formed between incomplete HER2-strong-positive and HER2-null cells | HER2 IHC staining slide |
| HER2strong_incomplete_HER2null_Degrees | Cellular connectivity | Average degree of incomplete HER2-strong-positive and HER2-null cells in graph network | HER2 IHC staining slide |
| HER2weak_complete_HER2weak_complete_minEdgeLength | Spatial distance | Minimum edge length between complete HER2-weak-positive and complete HER2-weak-positive cells | HER2 IHC staining slide |
| HER2weak_complete_HER2weak_complete_meanEdgeLength | Spatial distance | Mean edge length between complete HER2-weak-positive and complete HER2-weak-positive cells | HER2 IHC staining slide |
| HER2weak_complete_HER2weak_complete_Nsubgraph | Cluster topology | Number of subgraphs (clusters) formed between complete HER2-weak-positive and complete HER2-weak-positive cells | HER2 IHC staining slide |
| HER2weak_complete_HER2weak_complete_Degrees | Cellular connectivity | Average degree of complete HER2-weak-positive and complete HER2-weak-positive cells in graph network | HER2 IHC staining slide |
| HER2weak_complete_HER2weak_incomplete_minEdgeLength | Spatial distance | Minimum edge length between complete HER2-weak-positive and incomplete HER2-weak-positive cells | HER2 IHC staining slide |
| HER2weak_complete_HER2weak_incomplete_meanEdgeLength | Spatial distance | Mean edge length between complete HER2-weak-positive and incomplete HER2-weak-positive cells | HER2 IHC staining slide |
| HER2weak_complete_HER2weak_incomplete_Nsubgraph | Cluster topology | Number of subgraphs (clusters) formed between complete HER2-weak-positive and incomplete HER2-weak-positive cells | HER2 IHC staining slide |
| HER2weak_complete_HER2weak_incomplete_Degrees | Cellular connectivity | Average degree of complete HER2-weak-positive and incomplete HER2-weak-positive cells in graph network | HER2 IHC staining slide |
| HER2weak_complete_HER2null_minEdgeLength | Spatial distance | Minimum edge length between complete HER2-weak-positive and HER2-null cells | HER2 IHC staining slide |
| HER2weak_complete_HER2null_meanEdgeLength | Spatial distance | Mean edge length between complete HER2-weak-positive and HER2-null cells | HER2 IHC staining slide |
| HER2weak_complete_HER2null_Nsubgraph | Cluster topology | Number of subgraphs (clusters) formed between complete HER2-weak-positive and HER2-null cells | HER2 IHC staining slide |
| HER2weak_complete_HER2null_Degrees | Cellular connectivity | Average degree of complete HER2-weak-positive and HER2-null cells in graph network | HER2 IHC staining slide |
| HER2weak_incomplete_HER2weak_incomplete_minEdgeLength | Spatial distance | Minimum edge length between incomplete HER2-weak-positive and incomplete HER2-weak-positive cells | HER2 IHC staining slide |
| HER2weak_incomplete_HER2weak_incomplete_meanEdgeLength | Spatial distance | Mean edge length between incomplete HER2-weak-positive and incomplete HER2-weak-positive cells | HER2 IHC staining slide |
| HER2weak_incomplete_HER2weak_incomplete_Nsubgraph | Cluster topology | Number of subgraphs (clusters) formed between incomplete HER2-weak-positive and incomplete HER2-weak-positive cells | HER2 IHC staining slide |
| HER2weak_incomplete_HER2weak_incomplete_Degrees | Cellular connectivity | Average degree of incomplete HER2-weak-positive and incomplete HER2-weak-positive cells in graph network | HER2 IHC staining slide |
| HER2weak_incomplete_HER2null_minEdgeLength | Spatial distance | Minimum edge length between incomplete HER2-weak-positive and HER2-null cells | HER2 IHC staining slide |
| HER2weak_incomplete_HER2null_meanEdgeLength | Spatial distance | Mean edge length between incomplete HER2-weak-positive and HER2-null cells | HER2 IHC staining slide |
| HER2weak_incomplete_HER2null_Nsubgraph | Cluster topology | Number of subgraphs (clusters) formed between incomplete HER2-weak-positive and HER2-null cells | HER2 IHC staining slide |
| HER2weak_incomplete_HER2null_Degrees | Cellular connectivity | Average degree of incomplete HER2-weak-positive and HER2-null cells in graph network | HER2 IHC staining slide |
| HER2null_HER2null_minEdgeLength | Spatial distance | Minimum edge length between HER2-null and HER2-null cells | HER2 IHC staining slide |
| HER2null_HER2null_meanEdgeLength | Spatial distance | Mean edge length between HER2-null and HER2-null cells | HER2 IHC staining slide |
| HER2null_HER2null_Nsubgraph | Cluster topology | Number of subgraphs (clusters) formed between HER2-null and HER2-null cells | HER2 IHC staining slide |
| HER2null_HER2null_Degrees | Cellular connectivity | Average degree of HER2-null and HER2-null cells in graph network | HER2 IHC staining slide |
